# Formulating a Historical and Demographic Model of Recent Human Evolution Based on Resequencing Data from Noncoding Regions

**DOI:** 10.1371/journal.pone.0010284

**Published:** 2010-04-22

**Authors:** Guillaume Laval, Etienne Patin, Luis B. Barreiro, Lluís Quintana-Murci

**Affiliations:** 1 Human Evolutionary Genetics, Institut Pasteur, Paris, France; 2 Centre National de la Recherche Scientifique, URA3012, Paris, France; State University of New York College at Oneonta, United States of America

## Abstract

**Background:**

Estimating the historical and demographic parameters that characterize modern human populations is a fundamental part of reconstructing the recent history of our species. In addition, the development of a model of human evolution that can best explain neutral genetic diversity is required to identify confidently regions of the human genome that have been targeted by natural selection.

**Methodology/Principal Findings:**

We have resequenced 20 independent noncoding autosomal regions dispersed throughout the genome in 213 individuals from different continental populations, corresponding to a total of ∼6 Mb of diploid resequencing data. We used these data to explore and co-estimate an extensive range of historical and demographic parameters with a statistical framework that combines the evaluation of multiple models of human evolution via a best-fit approach, followed by an Approximate Bayesian Computation (ABC) analysis. From a methodological standpoint, evaluating the accuracy of the parameter co-estimation allowed us to identify the most accurate set of statistics to be used for the estimation of each of the different historical and demographic parameters characterizing recent human evolution.

**Conclusions/Significance:**

Our results support a model in which modern humans left Africa through a single major dispersal event occurring ∼60,000 years ago, corresponding to a drastic reduction of ∼5 times the effective population size of the ancestral African population of ∼13,800 individuals. Subsequently, the ancestors of modern Europeans and East Asians diverged much later, ∼22,500 years ago, from the population of ancestral migrants. This late diversification of Eurasians after the African exodus points to the occurrence of a long maturation phase in which the ancestral Eurasian population was not yet diversified.

## Introduction

The evolution, origins and geographic dispersals of modern humans remain among the most hotly debated issues in many disciplines, including paleoanthropology, archeology, linguistics and genetics. Roughly 100,000 years ago, the Old World was occupied by a morphologically diverse group of hominids: *Homo sapiens* in Africa and possibly the Middle East, Neanderthals in Europe and *Homo erectus* in Asia. However, by 25,000 years ago humans were present everywhere in the anatomically and behaviorally modern form. For the moment, the majority of anatomical, archaeological and genetic evidence support the view that modern humans are a recent species that originated in Africa and that subsequently replaced (mostly) existing hominid species in Europe and Asia [1]–[8]. Estimating the historical and demographic parameters that characterize modern human populations is a fundamental part of reconstructing human evolution [2]–[4]. Because past demographic events, such as changes in population sizes, geographic range expansions, and varying levels of gene flow, have produced specific patterns of genetic diversity, the study of genetic variation in present-day human populations allows inference of the general demographic models best explaining neutral genetic variability [9]. Furthermore, evaluation of these demographic scenarios is needed to disentangle the mimicking effects of population demography and natural selection on genome diversity [10]–[14]. In this context, the assessment of an appropriate neutral model of human evolution is required to identify confidently regions of the human genome that have been targeted by natural selection. This can in turn provide insights into human adaptive history, the mechanisms of evolutionary change, and potentially the identification of complex disease genes [9]. Understanding population variability under neutral conditions has therefore important implications in searching for genetic variants that might contribute to disease susceptibility [3], [13]–[15].

Efforts to reconstruct human origins and migration patterns have often focused on phylogeographic studies of the paternally inherited Y-chromosome and the maternally inherited mitochondrial DNA [16]–[18]. These studies have helped (i) clarifying the rough picture of human evolution (i.e., African origin of modern humans) [16], [19]–[23], (ii) unraveling the way modern humans spread around the world [17], [18], and (iii) unmasking sex-specific differences in migration rates and cultural practices [24]–[29]. However, due to the inherent properties of these two markers (e.g., single locus, low effective population size, uniparentally inherited), they provide a relatively partial model of human evolution. Multilocus autosomal studies based on single nucleotide polymorphisms (SNPs) [9], [30]–[32], short tandem repeats [33]–[37] or resequencing data [10], [38]–[44] have also provided new insights into recent human evolution. The advantage of resequencing studies, with respect to SNP data, is that they are free of ascertainment bias, allowing exploration of all aspects of genetic variation (e.g., low-frequency variants), and can be used in the context of statistical frameworks that make efficient use of most information contained in the data. Some of these resequencing studies have focused on gene regions and provided new insights into the effects of natural selection and human demography on genome diversity [10], [41], [42].

Few studies, however, have focused on resequencing regions of the genome specifically designed for demographic inference; segments that neither contain nor are tightly linked to coding regions [38], [40], [43], [44]. For example, one of these studies made use of the approximate likelihood approach for parameter estimation, based on summary statistics computed from 118 kb of sequence per individual from 45 individuals belonging to three different populations [40]. Another study used a Bayesian setting to analyze sequence diversity at 25 kb per individual in 30 individuals of African, Asian, and Native American origins [38]. Both studies estimated a number of demographic and historical parameters of recent human evolution. Because of the importance of jointly considering multiple parameters for reliable estimations [40], [45], we performed joint estimations (co-estimations) of all key historical and demographic parameters. For example, inter-continental migration, even if weak, has probably occurred, and neglecting this parameter in demographic inference may bias the estimation of other parameters (e.g. migration can diminish the signal of a bottleneck, see discussion of this point in the Results section).

Here we co-estimate multiple historical and demographic parameters of recent human evolution to provide an evolutionary model best explaining neutral genetic variability. We resequenced 20 independent noncoding autosomal regions dispersed throughout the genome, accounting for a total of 27 kb per individual, in a large population panel of 213 individuals from different continental populations, which may help to obtain a more general picture of human demographic history. To analyze this resequencing dataset (∼6 Mb of diploid noncoding resequencing data), we adopted an Bayesian setting, which is a convenient way to jointly estimate several parameters and therefore deal with the potential problem of inter-dependence among parameters [45]. We thus analyzed our data with simulation-based approaches [38], [46]–[49], which allowed us to jointly estimate multiple fundamental parameters of human evolution in a suitable computational time. Co-estimated parameters included historical parameters such as the time of both the out-of-Africa exodus and the split of the ancestral Eurasian population into current Europeans and East-Asians, as well as demographic parameters such as the effective population size of humans before the out-of-Africa exodus and of Eurasians after the bottleneck, the intensity of such a bottleneck, the onset and range of the African expansion(s), the effective population sizes of continental populations as well as the migration rates among them. All these co-estimations were jointly performed according to the most parsimonious set of historical and demographic assumptions in the best-fit model. In addition, we used a statistical framework that allowed us to formally test the accuracy of the parameter estimation and, most importantly, the sensitivity of these estimations to (i) the prior distribution of the estimated parameters, and (ii) the choice of the model of modern human dispersals out of Africa.

## Results

### Summary Statistics of Within- and Inter-Population Sequence Variation

We resequenced 20 independent, noncoding, autosomal regions in 213 individuals belonging to different continental groups, including 118 sub-Saharan African agriculturalists, 47 Europeans and 48 East-Asians individuals. The total length of sequence surveyed was ∼27 kb of diploid sequence per individual, with a mean length of ∼1.3 kb per genomic region (Table S1). The levels of nucleotide diversity observed are in good agreement with previous studies based on multi-locus re-sequencing [40] (Table 1), with average values of nucleotide diversity, ***π***, of 1.2×10^−3^ per nucleotide, with a between-region standard deviation of 0.63×10^−3^. The number of haplotypes and the levels of nucleotide diversity were the highest in the African sample, an observation that is expected under the out-of-Africa model (Table 1).

**Table 1:** Summary statistics for the 20 unlinked, noncoding autosomal regions.

Population	*K*		*S*		*π*		*D*		*Fs*		*F* *		*H*	
Sub-Saharan Africans	**15.15** ^***^	(5.04)	**14.2** ^***^	(5.35)	1.2×10^−3^	(0.6×10^−3^)	−0.85 ^***^	(0.63 *)	−5.75 ^***^	(3.94)	−1.75	(1.3)	−0.25	(0.76)
Europeans	6.1	(1.59 ^**^)	6.7	(2.52 ^**^)	1.0×10^−3^	(0.7×10^−3^)	0.1	(1.14)	0.25	(2.4)	−0.07	(1.26)	−0.24	(0.83)
East-Asians	5.3	(1.59 ^**^)	5.75	(2.61 ^**^)	0.9×10^−3^	(0.6×10^−3^)	0.1	(1.08)	0.24	(2.47)	0.06	(1.25)	−0.66 ^**^	(1.19)

Note.– *K* denotes the number of haplotypes, *S* denotes the number of polymorphic sites, *π* denotes the nucleotide diversity, *D* denotes the Tajima's *D* statistics, *Fs* denotes the Fu's Fs statistics, F* denotes the Fu and Li's F* statistics, and *H* denotes the Fay and Wu's *H* statistics. The summary statistics were averaged over the 20 unlinked autosomal regions and the standard deviations are given in parentheses. Significant deviations (Material and Methods) from a model with constant population size are indicated in bold when values are significantly increased, and underlined when values are significantly reduced.

* *P*<0.05, ^**^ *P*<0.01 and ^***^ *P*<0.001, using the most conservative *P*-value (the highest *P*-value) among the several recombination rates used in the simulations (Materials and Methods, Table S2).

**Figure 1:**
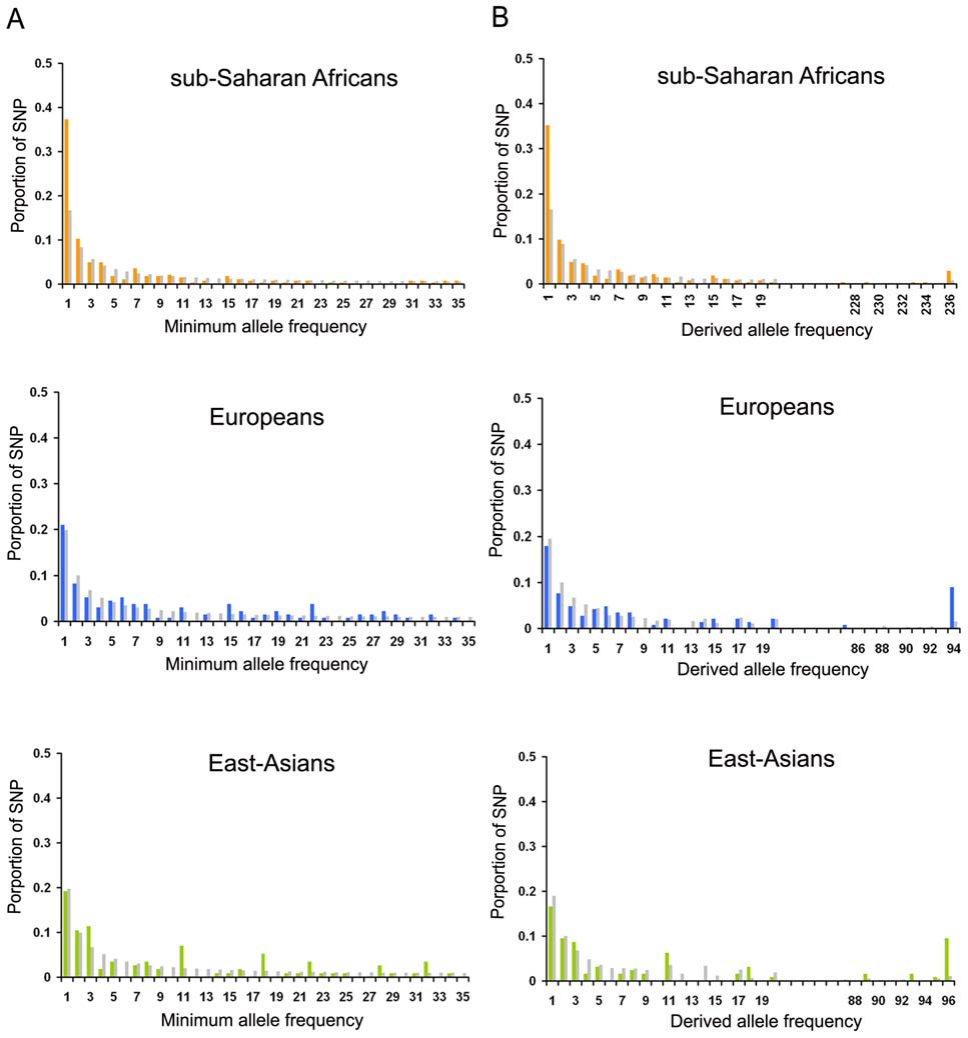
Minor allele and derived allele frequency spectra. (**A**) Minor allele frequency (MAF) and (**B**) derived allele frequency (DAF) spectra computed by merging the 20 non coding autosomal DNA sequences. The expected MAF and DAF spectra (grey bars) were obtained assuming constant population sizes (Material and Methods). To focus on low frequency bins, the MAF spectrum display values lower than 35 counts in each continental population. To show the derived alleles that are fixed in each continental population, we arbitrarily removed intermediate bins in the DAF spectrum.

**Figure 2:**
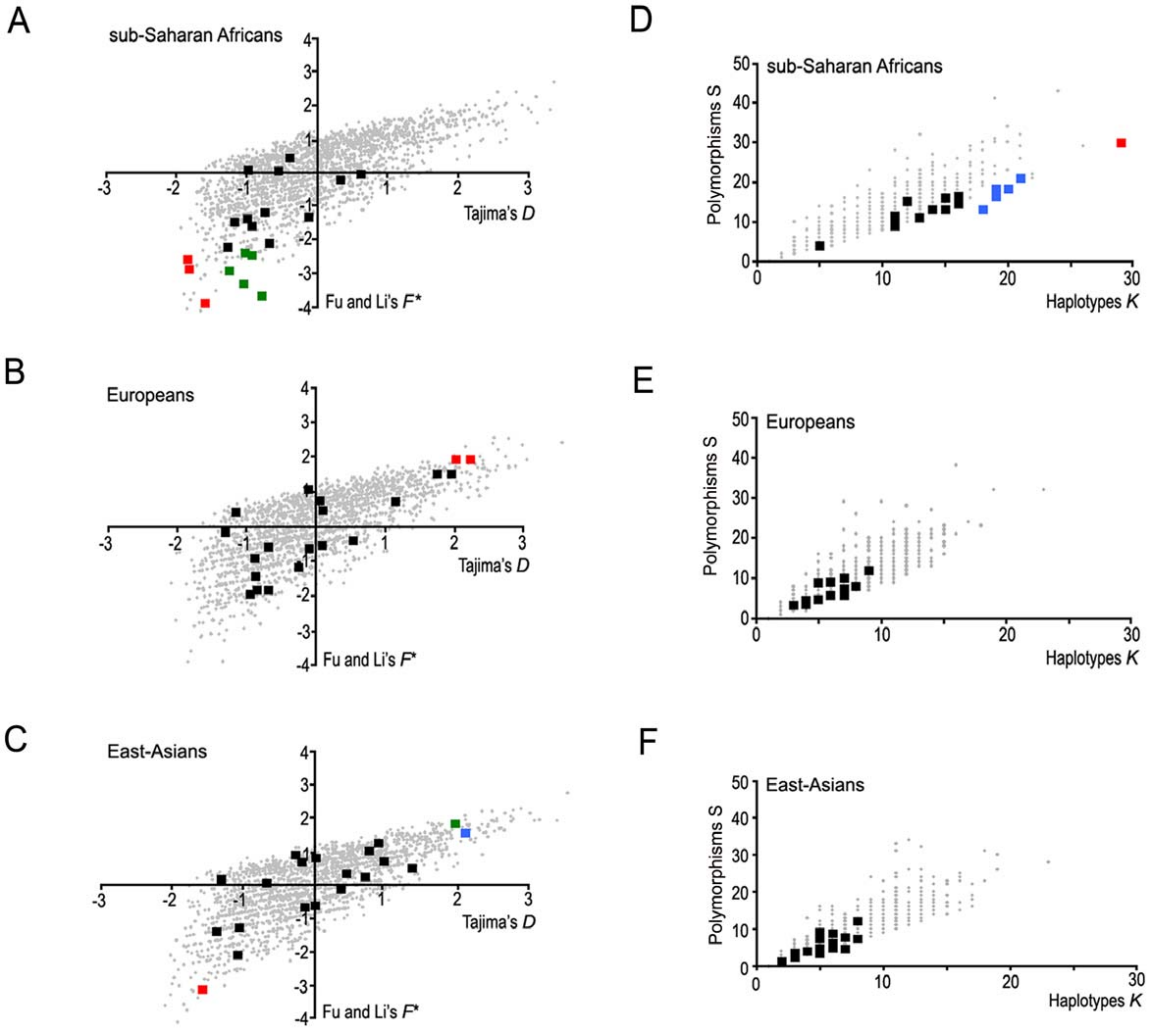
Sequenced-based summary statistics in Africans, Europeans and East-Asians. Biplots of Tajima's *D* and Fu and Li's F* computed for each genomic region separately, in Africans (A), Europeans (B) and East-Asians (C). Significant Tajima's *D* values (*P*<0.05) are indicated in blue, in green for Fu and Li's F* only, and in red for both. Biplots of the number of haplotypes (*K*) and polymorphisms (*S*) computed for each genomic region separately in Africans (D), Europeans (E) and East-Asians (F). Significant *K* values (*P*<0.05) are indicated in blue, in green for *S*, and in red for both. The grey dots indicate the expected values of each genomic region simulated assuming a constant population size model (simulation procedure and significance of each region are described in the Materials and Methods section).

To test for deviation from the “null model” (i.e., a model involving a constant-sized population), we computed a number of statistics summarizing several aspects of the data. First, we computed the minor allele frequency (MAF) spectrum and the derived allele frequency (DAF) spectrum (Figure 1). In the sub-Saharan African sample, both the MAF and the DAF spectra showed a highly significant increase in the proportion of singletons with respect to the proportion expected under a constant population size model (χ^2^ *P* = 3×10^−8^ and χ^2^ *P* = 9×10^−5^, respectively). In addition, eight of the twenty genomic regions studied showed significantly negative values of Tajima's *D* or Fu and Li's F* (Figure 2A), leading to a significantly negative mean of Tajima's *D* value across the 20 regions. The mean of Fu's *Fs* across the twenty regions was also negative and highly significant (Tables 1 and S2). In addition, six regions exhibited a significant increase in the number of haplotypes (Figure 2D), and when averaging the values across regions, both a significant increase in the number of haplotypes and polymorphic sites were observed, with respect to expectations under a model of constant-population size (see Materials and Methods, and Tables 1 and S2). Altogether, these patterns strongly support the occurrence of at least one phase of population expansion among sub-Saharan Africans. With respect to Eurasian samples, we observed an excess of derived allele frequencies that reached fixation in European and East-Asian samples (χ^2^ *P* = 4×10^−3^ and χ^2^ *P* = 2×10^−3^, respectively) (Figure 1B). These results support the hypothesis that European and East-Asian populations may have experienced one or several bottlenecks. Although most sequence-based neutrality statistics did not significantly deviate from neutral expectations (except for the negative value of Fay and Wu's *H* in East-Asians and a few single statistics when analyzing the genomic regions separately, see Tables 1 and S2, Figures 2B, C, E and F), the between-region standard deviations of the number of haplotypes and polymorphic sites were significantly reduced (Tables 1 and S2). These features are also expected after a bottleneck (Figure S1).

With respect to inter-population diversity, our multi-ethnic panel showed levels of population differentiation similar to those previously observed [50], with a significant global *F*_ST_ (merging all samples) averaged over the 20 genomic regions equal to 0.12. Pairwise *F*_ST_ among the five sub-Saharan African populations were not significantly different from 0, and pairwise *F*_ST_ between Danes and Chuvash and between Han Chinese and Japanese were weak (*F*_ST_ = 0.01 and *F*_ST_ = 0.03, respectively) (Table S3).

### Best-Fit of Human Demography

To identify a relevant historical and demographic model characterizing modern human populations, we first sought to reduce the space of models and parameters to explore by using a model-fitting approach, and then co-estimate parameters within the best-fit model using an Approximate Bayesian Computation (ABC) framework. We divided the first step (i.e. the definition of a general best-fit model of modern human history) into two parts: we first tested different models defined by fluctuating levels of structure and gene flow in the ancestral population, prior to the appearance of modern humans. We then tested different models defined by fluctuations of the effective size of each continental population of modern humans. For all the best-fit procedure, we simulated each alternative scenario 10^5^ times and compared the simulated statistics to the observed statistics computed from our empirical dataset (20 re-sequenced regions). All parameters used to simulate the different scenarios were randomly drawn from distributions presented in Table S4.

**Figure 3:**
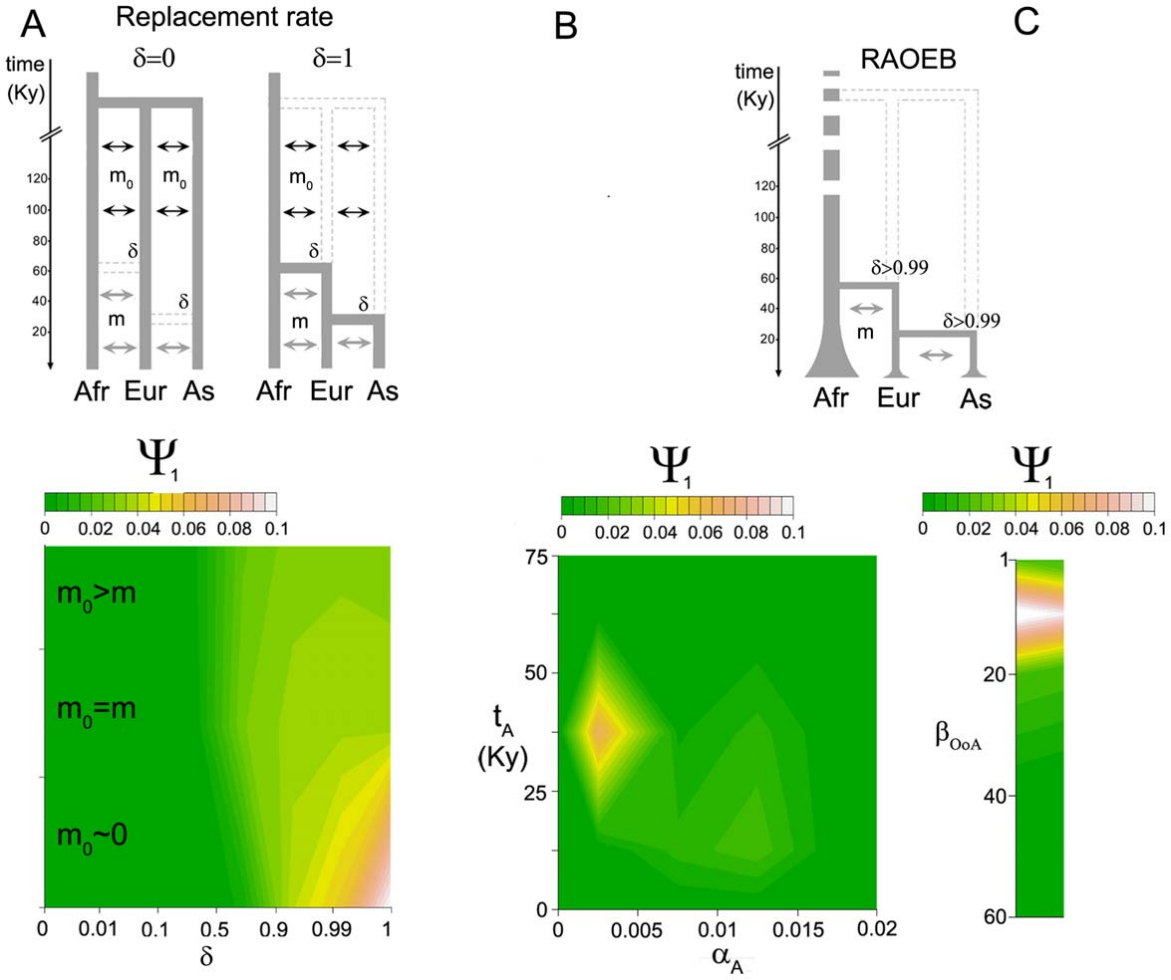
Model and parameter best-fitted estimations. (**A**) Simulations considering different levels of replacement of archaic hominids by modern humans. We performed 8 sets of 10^5^ simulations: one set for a replacement rate ***δ*** = 0, one for ***δ*** = 1, 3 sets for 0≤***δ***≤0.01, 0≤***δ***≤0.1 and 0≤***δ***≤0.5, and 3 sets for ***δ***≥0.5, ***δ***≥0.9 and ***δ***≥0.99. For each of the 8 sets, we considered three models of ancestral migration (represented by black arrows): a residual ancestral migration rate (***m_0_***∼10^−10^), an ancestral migration rate with the same range (10^−6^ to 4×10^−3^) as ***m*** the current migration rate (represented by gray arrows), and an ancestral migration twice higher than ***m***. Among the 24 models tested, the model assuming a complete replacement rate of archaic hominids (δ = 1) and a residual ancestral migration (***m_0_***∼10^−10^) exhibited the significantly highest ψ_1_ except when compared with the model assuming an almost complete replacement rate of archaic hominids (δ≥0.99). This best-fitted range of parameters (***δ***≥0.99 and ***m_0_***∼10^−10^), indicated by the yellow/orange/white area (**A**), was therefore used to simulate the African expansion (**B**) and the non African bottleneck (**C**). We performed three sets of 10^5^ simulations for the onset ***t_A_***: 0≤***t_A_***≤25 Kyears, 25≤***t_A_***≤50 Kyears and 50≤***t_A_***≤75 Kyears. For each of the three sets, we considered 5 models of growth rate ***α_A_*** parameters; ***α_A_*** = 0, 0≤***α_A_***≤0.005, 0.005≤***α_A_***≤0.01, 0.01≤***α_A_***≤0.015 and 0.015≤***α_A_***≤0.02. Among the 15 models tested, the best-fitted ranges of parameters (ψ_1_ significantly higher than ψ_1_ of the constant size model ***α_A_*** = 0, P<0.01) are indicated by the yellow/orange/white area (**B**). Likewise, we performed 5 sets of 10^5^ simulations assuming bottlenecks intensities ***β_OoA_***, starting at the time of the out-of-Africa exodus (***T_OoA_***) and ending at the independent Neolithic expansions in Europe and east-Asia: ***β_OoA_*** = 1, 1≤***β_OoA_***≤2, 2≤***β_OoA_***≤20, 20≤***β_OoA_***≤40 and 40≤***β_OoA_***≤60. The best-fitted range of parameter (ψ_1_ significant higher than ψ_1_ of the constant size model ***β_OoA_*** = 1, P<0.01), indicated by the yellow/orange/white area (**C**), was obtained with the set of priors 2≤***β_OoA_***≤20. The distributions used are specified in Table S4.

First, we determined the evolutionary scenario that took place in the ancestral lineage that culminated in the emergence of modern humans (for a complete list of parameter symbols used along the manuscript, see Tables 2 and S4). We tested different evolutionary models [2], [5], [19], [22], [51]–[56] that allow different levels of introgression of archaic hominids to modern human populations. We assumed an early diffusion of archaic hominids (*Homo erectus*) out of Africa ∼1.25 and ∼2.25 million years ago [57], various ancestral migration rate intensities (***m_0_***, ancestral migration rate is the proportion of migrants before the Out-of-Africa exodus) and an African exodus of modern humans between ∼40,000–100,000 years ago [38]. By tuning the replacement rate ***δ***, we then simulated scenarios that consider different levels of replacement of archaic hominids by modern humans (i.e. different levels of introgression of archaic material into the modern gene pool), including the most extreme cases of complete (***δ*** = 1) and no replacement (***δ*** = 0) as well as several scenarios with varying intermediate levels of replacement (Figures 3A and S2, Table S4). The summary statistics were calculated by merging all population samples (except for global *F*_ST_) in order to minimize the effects of recent demographic events related to the continental populations. We thus considered in all models a constant size for the three modern human populations. The model with residual ancestral migration rate (***m_0_***∼10^−10^) and full replacement (***δ*** = 1) clearly better fitted our data than any other model (Figure 3A, highest ψ_1_, the ψ_1_ of this model is significantly higher after correction for multiple testing when compared with the other ψ_1_ values, P<0.01). However, we could not discern between a complete (***δ*** = 1) and an almost-complete (***δ***≥0.99) replacement of archaic hominids (difference between ψ_1_ is not significant for this pairwise comparison), indicating that a small contribution of archaic humans to our present-day genome cannot be completely ruled out [58]–[61].

**Table 2:** Prior distributions of the parameters for the best-fit (RAOEB) model.

Parameters		mean	min	Max	Shape^a^
Exit of archaic humans from Africa	*T_E_* b	1.9×10^6^	1.2×10^6^	2.5×10^6^	∼U
**Modern humans African expansion**					
Onset of African expansion	*t_A_* b	17750	5000	50000	∼U^c^
Rate of African expansion	*α_A_* d	0.009	0.002	0.02	∼U^c^
Ancestral African effective population size	*N'* e	10000	500	40000	∼G
African effective population size	*N_A_* e	3.3×10^7^	1500	10^9^	ND
**Non African bottleneck**					
Exit of modern humans from Africa	*T_OoA_* b	66260	45020	87500	∼U
Population size after out-of-Africa exodus	*N_OoA_* e	850	51	24000	ND
Intensity of out-of-Africa bottleneck	*β_OoA_* f	15	1	30	∼U
Onset of Neolithic expansion in Europe	*t_E_* b	8750	5000	12500	∼U
Rate of Neolithic expansion in Europe	*α_E_* d	0.00255	0.0001	0.005	∼U
European effective population size	*N_E_* e	5000	50	150000	ND
Onset of Neolithic expansion in East-Asia	*t_EA_* b	8750	5000	12500	∼U
Rate of Neolithic expansion in East-Asia	*α_EA_* d	0.00255	0.0001	0.005	∼U
East-Asian effective population size	*N_EA_* e	5000	50	150000	ND
**Migration among populations**					
Modern human migration rate between continents	*m* g	2×10^−4^	10^−6^	4×10^−3^	ND
Ancestral migration rate	*m_0_* g	1.7×10^−10^	10^−11^	4×10^−9^	ND
**Non-African historical parameters**					
Replacement rate	*δ* h	0.995	0.99	1	∼U
Time of European/East-Asian split	*T_E-EA_* b	25010	12520	37500	∼U
**DNA features**					
Mutation rate	*μ* i	2.5×10^−8^	1.3×10^−8^	5×10^−8^	∼G
Recombination rate	*ρ* j	10^−8^	0.1×10^−8^	1.5×10^−8^	∼G

a ∼U and ∼G denote Uniformly and Gamma distribution shapes. ND (for not drawn) indicates composite parameters resulting from the combination of other parameters (e.g. the Sub-Saharan African population size results from the combination of *N'*, *t_A_* and *α_A_*);

b Times *T* and onsets *t* are expressed in number of years (generation times of 25 years);

c Prior distributions of the onset and the rate of African expansion were set to prior uniform distributions (unrealistic outcomes of sub-Saharan African populations, i.e. larger than 1 billion of individuals were eliminated);

d The rates of expansion *α* are the per generation increase of population sizes expressed in percent of individuals (i.e. *α_A_* = 0.01 means the population exponentially increased by 1% of the individuals per year);

e Effective population sizes *N* are given in numbers of individuals;

f The intensity of the out-of-Africa bottleneck is the ratio between population sizes before and after the out-of-Africa exodus;

g ancestral and modern migration rates are the proportion of migrants before and after the Out-of-Africa exodus;

h The replacement rate gives the proportion of current gene lineages brought by modern humans during the out-of-Africa exodus;

i The mutation rate is expressed in per generation per site; and

j the recombination rate is expressed in per generation per pair of adjacent bases. Note: Underlined parameters were estimated following the ABC procedure.

We tested the extent to which the choice of this evolutionary model is robust to potential differences among models tested (e. g. different numbers of parameters, etc.) and to the high variability of datasets that can be generated by a given evolutionary scenario. To this effect, we simulated 100 pseudodatasets under the best-fit model (highest *ψ_ξ_* obtained using our actual empirical dataset) and the other alternative models. We first performed pairwise comparisons between the best-fit model (residual ancestral migration and nearly full replacement, ***δ***≥0.99) and the minor replacement (***δ***≤0.5) models (Figure 3A). Independently of the values of replacement rate (***δ***) and ancestral migration rate (***m_0_***) considered, we found that our approach identifies the “correct” model in more than 98% of the cases (out of the 200 pseudodatasets simulated for each pairwise comparison, see Materials and Methods for a full explanation). We next compared this best-fit model (residual ancestral migration and nearly full replacement, ***δ***≥0.99) with other models involving major replacement (***δ***≥0.5, Figure 3A), and we found that, independently of the values of ancestral migration rate (***m_0_***), our approach still identifies the “correct” model in more than 95% of cases (200 simulated pseudodatasets for each pairwise comparisons). The only exception found concerns the comparison between the best-fit model (***δ***≥0.99) and the model with residual ancestral migration and a strong replacement (***δ***≥0.9, Figure 3A). In this case, we obtained 65% of correct model assignation over the 200 pseudodatasets used, confirming the difficulty in discriminating between values of ***δ*** that reflect high levels of replacement of archaic humans in Eurasia.

**Figure 4:**
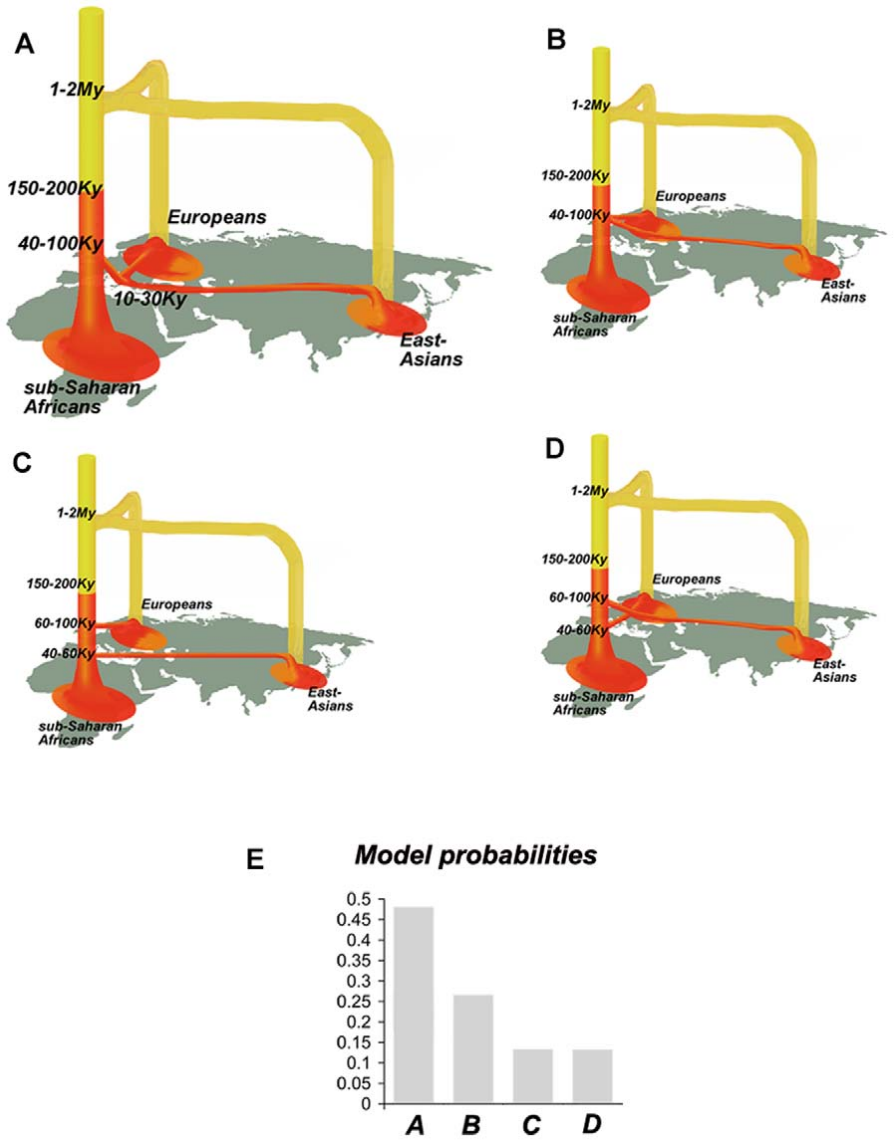
Models of recent African origin involving different dispersal scenarios. (**A**) General RAOEB model best fitting the data, with parameter ranges given in Table 2. This model assumes a single out-of-Africa dispersal followed by the European and East-Asian split. (**B**) RAOEB model involving two independent, concomitant dispersals out of Africa, each giving rise to Europeans and East-Asians. (**C**) RAOEB model involving two independent dispersals out of Africa occurring at different times, the earlier giving rise to Europeans. (**D**) RAOEB model involving two independent dispersals out of Africa occurring at different times, the earlier giving rise to East Asians. For models **B–D**, the ranges of parameters are the same as those given in Table 2. The alternative dispersal model **B** (two independent dispersals at the same time) was performed using a split of the two non Africans populations concomitant with the time of out-of-Africa exodus (***T_OoA_***) simulated with the same prior reported in Table 2. The two alternative dispersal models **C** and **D** (two independent dispersals at different times) were simulated using times for the first out-of-Africa exodus drawn from the first half of the prior distribution of ***T_OoA_*** (Table 2), while times for the second out-of-Africa exodus were drawn from the second half of the prior distribution of ***T_OoA_***. (**E**) Posterior probability estimated for the 4 possible dispersal models represented in A, B, C, and D.

We next refined this best-fit model (i.e. ***m_0_***∼10^−10^, δ≥0.99) by testing for the demographic history of each continental group (Figures 3B–C). Specifically, we investigated the local demographic history (population growth, bottleneck events), by using a set of summary statistics averaged over the 20 genomic regions, for the three continental groups separately (Table 1). We simulated a scenario that included various demographic events (i.e. African expansion and non-African bottleneck models, Table S4), that may have generated the significant deviations from the constant-sized model observed in the summary statistics (Table 1). With respect to African populations, we tested for the occurrence of varying onsets (***t_A_***) and intensities (***α_A_***) of population expansion including the constant size model (***α_A_*** = 0) (Figure 3B). Models involving an expansion at 25,000–50,000 years were those best supported by the data (Figure 3B, highest ψ_1_, the only significant comparison after correction for multiple testing when all values of ψ_1_ are compared with the ψ_1_ of the constant size model, P<0.01). This result confirms the classical neutrality tests, which already supported population growth in Africa by rejecting the constant size model (e.g. significantly negative Tajima's D in Figure 2A, Tables 1 and S2). With respect to non-African populations, we tested for the occurrence of bottlenecks of varying intensities (***β_OoA_***, being the ratio between the population sizes before and after the bottleneck event), including the constant size model (***β_OoA_*** = 1) (Figure 3C). The model that best fitted our data involves a substantial bottleneck among non-Africans (Figure 3C, 2≤***β_OoA_***≤20 giving the highest ψ_1_ and the only significant comparison after correction for multiple testing when all values of ψ_1_ are compared with the ψ_1_ of the constant size model, P<0.01), rejecting significantly a constant population size model for these populations. Taken together, this best-fitted model (Figure 4A) is consistent with the family of proposed out-of-Africa models [9], [35], [38] and supports the occurrence of population growth among sub-Saharan Africans and a bottleneck among non-Africans [39], [40]. In what follows, we will refer to this model as to the “RAOEB” model (i.e. Recent African Origin with Expansion and Bottleneck”).

By comparing this best-fitted continental demographic scenario with other alternative models with varying parameters of the African expansion (Figure 3B) and the non-African bottleneck (Figure 3C), we found that our approach identifies the “correct” model in (i) more than 90% of the cases between the best-fitted African expansion and other expansion alternatives (200 simulated pseudodatasets for each pairwise comparison), and (ii) more than 99% of the cases between the best-fitted non-African bottleneck and other bottleneck alternatives (200 simulated pseudodatasets for each pairwise comparison).

### Co-Estimating Historical and Demographic Parameters under the RAOEB Model

The parameters ranges obtained using the best-fit approach (1^st^ step, Figure 3B–C) were obtained under non-optimal conditions, that is, considering independently the African expansion and the non-African bottleneck. Indeed, the co-estimation of the different demographic parameters is necessary to provide consistent estimations. For example, different rates of migration (i.e., gene flow) can mimic different degrees of population expansion (Figure S3), and this can affect the accuracy of the estimations (e.g. underestimation of the intensity of a bottleneck). Furthermore, little is known about the historical degree of inter-continental migration, for example, highlighting the need of methods able to estimate jointly all parameters (e.g. migration, bottleneck, expansion) because they are evolutionarily inter-dependent. We therefore co-estimated the historical and demographic parameters by using the ABC statistical framework (2^nd^ step) [45]–[47], [49]. Note that the 1^st^ step approach (definition of a best-fit model) allowed us to avoid the exploration of a wide range of unlikely parameter values in the 2^nd^ step approach (ABC co-estimation). Specifically, we considered residual ancestral migration (i.e. ***m_0_***∼10^−10^) and an almost-complete replacement of archaic hominids by excluding values of the replacement rate (***δ***) lower than 0.99. With respect to African populations, we excluded expansion rates values near to the constant size assumption (***α_A_***<0.002) since both classical neutrality tests (Table 1) and the best-fit approach (1^st^ step) confirmed that African populations have experienced an expansion. We also excluded values of rates (***α_A_***) and onsets (***t_A_***) of the African expansion found to be unrealistic, i. e. ***α_A_*** higher than 0.02 and ***t_A_*** older than 50,000. With respect to non-African populations, we excluded bottlenecks intensities (***β_OoA_***) higher than 30. In order to be cautious, the prior distributions used in the ABC estimation were slightly enlarged with respect to those obtained in the best-fit approach (i.e. calibrated under non-optimal conditions). Furthermore, we tested the influence of the calibrated prior distributions (Table 2) on ABC estimations by further extending them, mainly for parameters such as the onset and rate of African expansion, the ancestral African effective population size and the time of the out-of-Africa exodus (see below, section entitled “Investigating the accuracy of parameter co-estimation”).

**Figure 5:**
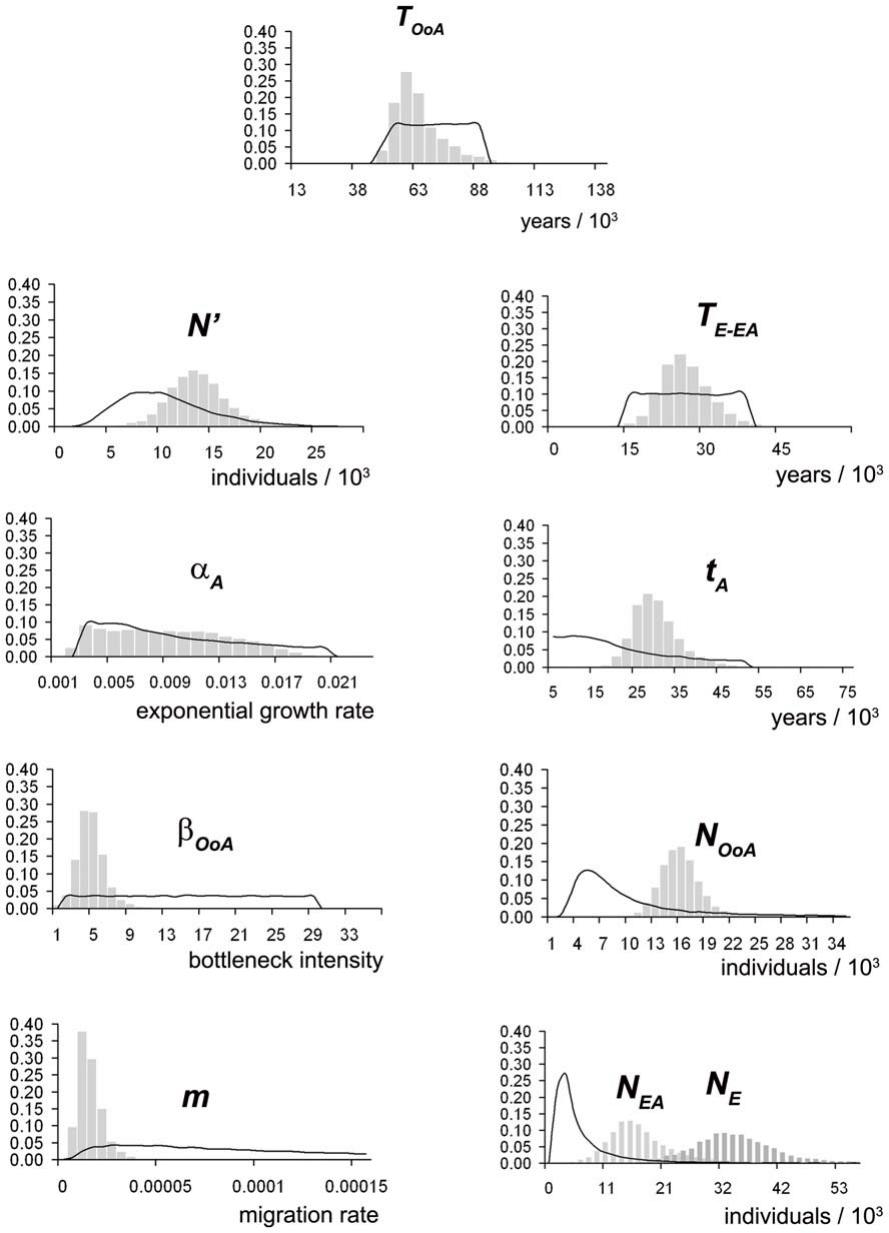
Approximate posterior distributions of historical and demographic parameters. This figure gives the estimated ABC posterior distributions of the historical and demographic parameters (Table 3) using the RAOEB model (Figure 4A) with best-fitted priors (Table 2). Black lines represent the prior distributions and grey bars the posterior distributions. The times were translated into years using a generation time equal to 25 years. The posterior distributions of the parameters where the estimations were not validated by means of the accuracy evaluation procedure are not presented (i.e. ***N_A_*** and ***δ***).

We performed 10^6^ simulations of the 20 genomic regions, using first the prior distributions given in Table 2, to estimate (i) historical parameters such as the time of the out-of-Africa exodus, ***T_OoA_***, the replacement rate, ***δ***, and the time of the subsequent European/East-Asian split, ***T_E-EA_***, and (ii) demographic parameters such as the effective population size of humans before the out-of-Africa exodus, ***N'***, the effective population size of Eurasians after the out-of-Africa exodus, ***N_Oo_***_*A*_, the effective population sizes of Africans (***N_A_***), Europeans (***N_E_***), and East-Asians (***N_EA_***), the onset, ***t_A_***, and the rate, ***α_A_***, of the African expansion, the intensity of the out-of-Africa bottleneck, ***β_OoA_***, and the migration rate among continental groups, ***m*** (Table 2). The co-estimations of all these parameters are shown in Table 3 and the corresponding posterior distributions in Figure 5. Our estimations (95% Bayesian confidence interval [CI] given in Table 3) indicated that modern human populations left Africa between 47,500 and 85,000 years ago, more probably 60,000 years ago. The exodus from an ancestral African population of ∼13,800 individuals left a signature in the genome of Eurasians equivalent to an exit out-of-Africa of 2,100 to 3,800 individuals. This bottleneck corresponds to a reduction of 2.6 to 8.8 times the effective population size, more probably 5.1. Following the early colonization of Eurasia, the ancestors of modern Europeans and East-Asians diverged from the population of ancestral migrants ∼22,500 years ago (95% CI 17,500–35,000 years ago), leading to effective population sizes estimated at ∼31,200 and ∼14,500 individuals in Europe and East Asia, respectively. Concomitantly, African populations experienced an expansion that left a signature in their current genome compatible with an exponential demographic growth starting ∼27,500 years ago (95% CI 20,000 to 40,000 years ago) with a rate of 0.007 (95% CI 0.002 to 0.016) individuals per generation. In addition, inter-continental symmetric migrations occurred for an estimated 1.3×10^−5^ (95% CI 3.5×10^−6^ to 2.6×10^−5^) individuals per generation.

**Table 3:** Historical and demographic parameters estimated under the favored RAOEB model.

	Estimation^a^		Accuracy tests			
	*Estimate*	*95% CI* b	*B* c	*SE* d	*RMSE* e	*CI_hits_* f
***t_A_***	27500	20000–40000	0.03	0.42	0.42	0.96
***α_A_***	0.007	0.002–0.016	0.34	0.49	0.59	0.96
***N'***	13800	9000–19800	−0.04	0.31	0.31	0.96
***N_A_***	2.3×10^7^	6×10^5^–1.9×10^9^	−2.8	7.7	8.2	0.98
***T_OoA_***	60000	47500–85000	−0.01	0.17	0.17	0.98
***N_OoA_***	2800	2100–3800	0.00	0.20	0.20	0.98
***β_OoA_***	5.1	2.6–8.8	−0.14	0.40	0.42	0.97
***N_E_***	31200	19600–52100	−0.06	0.38	0.39	0.98
***N_EA_***	14500	7100–37900	−0.05	0.56	0.57	0.96
***m***	1.3×10^−5^	3.5×10^−6^–2.6×10^−5^	−0.05	0.31	0.32	0.97
***δ***	0.9949	0.9900–0.9997	−1 10^−5^	0.027	0.027	0.98
***T_E-EA_***	22500	17500–35000	0.00	0.24	0.24	0.97

a For each parameter estimate, we report the values obtained using the set of summary statistics (Table S10) giving the best accuracy (parameters in bold in Table S5);

b 95% Bayesian confidence interval estimated from posterior distributions;

c *B* is the average relative bias (standardized by the known parameter value);

d *SE* is the relative standard error (standardized by the known parameter value);

e *RMSE* is the relative root of mean square error (standardized by the known parameter value);

f *CI_hits_* is the percent of known values falling within the range of the 95% CI of the estimation.

### Investigating the Accuracy of Parameter Co-estimation

We next investigated the degree of accuracy of ABC parameter estimations. To this end, we simulated 100 pseudodatasets under the favored RAOEB model. For each of them, we re-estimated the underlying parameters using the same ABC procedure used for our empirical dataset. This approach allows comparison of parameter estimates with the known parameter values and provides several indexes of estimation accuracy (i.e. the bias, *B*, the standard error, *SE*, the root of mean square error, *RMSE*, and the percent of known values falling within the range of the 95% CI of the estimation, *CI_hits_*, see Material and Methods for details). We calculated these accuracy indexes for different sets of summary statistics (Table S5). Among these different sets of summary statistics, we selected for each parameter (Table 3) the set giving the best accuracy, i. e. lowest *RMSE*, (values in bold in Table S5, all parameter estimations using the different sets of statistics in Table S6). Generally, the average relative biases of parameter estimations were small (<5% of the known parameter value, with *RMSE* close to *SE*, which is a property of unbiased estimators) (Table 3). The relative standard errors were lower than 1 and generally close to 0.5 (*SE*<0.5 means ∼80% of the estimated values have a relative bias <50% of the known value). A marked exception to the generally good accuracy of our parameter estimations was the sub-Saharan African effective population size, ***N_A_***, which exhibited higher values of *B*, *SE*, and *RMSE* (Table 3). It is also worth mentioning that the replacement rate parameter, ***δ***, showed low *RMSE*, which could attest to a good estimation of this parameter. However, the range of variation of ***δ*** (prior distribution) is, in contrast to the others parameters, smaller than the simulated values (0.99<***δ***<1, range ∼1% of the value of ***δ***).

We next investigated the extent to which changing the shape of the priors and extending the range of their distributions could alter our parameter estimations (Table 3). The re-estimated parameter values as well as the shape of their posterior distributions (Figure S4, Table S7) were found to be robust to prior modulations. In addition, altering the prior shape for key parameters – such as the ancestral effective population size of humans (before the out-of-Africa exodus) ***N'*** – did not alter co-estimations of the remaining historical and demographic parameters (Table S8). The only parameter found not to be robust to prior modification was the replacement rate, ***δ***, preventing us to obtain reliable estimates for this parameter. However, and interestingly, this prior modification of ***δ*** did not alter the estimation of the remaining parameters (Table S8).

### Investigating the out of Africa Models of Dispersal(s)

We finally investigated the mode in which the different population dispersals out of Africa occurred to colonize Eurasia, by relaxing the assumption of single major dispersal event followed by the Eurasian split (Figure 4A). To this end, we simulated three additional models constituting different variants of the more general RAOEB model, involving (i) two independent and concomitant dispersals out of Africa, each giving rise to Europeans and East-Asians (Figure 4B), (ii) two independent dispersals out of Africa occurring at different times, the earlier giving rise to Europeans (Figure 4C), and (iii) two independent dispersals out of Africa occurring at different times, the earlier giving rise to East-Asians (Figure 4D). We merged the simulations made for each of the four alternative RAOEB models (Figure 4A–D) with the same probability each and using the prior distributions reported in Table 2. We used this composite simulated dataset of 10^5^ simulations to evaluate the posterior probability of each of the four alternative models within the general RAOEB model (Figure 4A–D). This was performed by using an additional parameter with 4 possible issues, each of them corresponding to a given model. We estimated the posterior probabilities of each of these 4 possible models by using the proportion of the simulations that best fit the data (5,000 smallest distances between simulated and empirical summary statistics, Φ parameter before regression as defined in [46]). Among these smallest distances, ∼50% of them (Figure 4E) corresponded to simulations of the model involving a single, major dispersal out of Africa followed by the Eurasian split (Figure 4A). In addition, we jointly re-estimated the posterior distributions of the historical and demographic parameters of the composite simulated dataset using the ABC approach. Importantly, the estimates (Table S9) and the related posterior distributions (Figure S5) obtained when merging these four alternative models (Figure 4A–D) are consistent with those previously obtained assuming a single dispersal event (Figures 4A and 5, Table 3). Therefore, the parameter estimates reported when assuming a single dispersal only are robust and not sensitive to the choice of the model of human dispersals out of Africa.

## Discussion

The study of the mode in which modern humans originated and colonized the world has important implications in questions of paleoanthropological interest but also in medical, epidemiological and population genetics. Here, we focused on the demographic processes that accompanied the global diaspora of modern humans after their origin in Africa. These processes include, among others, the time at which the African exodus of modern humans occurred, the intensity of the corresponding bottleneck, the sizes of the ancestral populations and how they expanded demographically, the extent to which modern humans replace archaic forms, and the way the different modern continental populations diverged from each other. To this end, we explored an extensive range of historical and demographic parameters characterizing recent human evolution using a statistical framework that combines multiple facets of the genetic data. Our approach combines the evaluation of different demographic models using a best-fit approach, followed by an ABC analysis of the data that conveniently deals with the co-estimation of multiple inter-dependent parameters [45], [46].

For those historical and demographic parameters that have been previously studied, our co-estimations are in agreement with previous reports, highlighting the general accuracy of our estimates. For example, our estimation of the replacement rate of archaic hominids by modern humans, although indicating that the introgression of archaic material into the gene pool of modern humans has been minimal, did not rule out the presence of minor archaic admixture of other hominids in modern humans in agreement with previous observations [58]–[61]. However, it is important to emphasize that our inferences are based on non-coding neutral regions of the genome and that adaptive introgression from archaic to modern humans may have occurred to a greater extent [62]. Indeed, in contrast to neutral alleles, adaptive variants may attain high frequencies by natural selection after minimal genetic introgression. Future studies comparing coding-sequence variation in modern humans and extinct hominids (e.g. Neanderthals) should help to answer this question. With respect to the time of the exit of modern humans out of Africa, our estimates (∼60,000 years ago) well match archeological records as well as molecular data [7], [8], [21], [23], [34], [38], [63]–[65]. The estimation of effective population sizes before (∼13,800) and after (∼2,800) the out of Africa exodus indicates a massive reduction (∼80%) of the effective population size during the bottleneck event, in agreement with the parameter ranges estimated from non-coding resequencing data [40]. In addition, our data is compatible with stronger genetic drift among East Asians than Europeans (***N_E_***>***N_EA_***) [30]. Most importantly, our analytical approach improved the inferences about past human demography for certain critical aspects of human demographic history. Our analyses support strong population growth among African populations 20,000–40,000 years ago, involving 0.002–0.016 individuals per generation. Our sub-Saharan African data – based on 118 individuals from 5 different agriculturalist populations spread over the African continent (Nigeria, Cameroon, Gabon, Tanzania and Mozambique) – extend previous claims of population growth based on single African populations to most of the African continent. Whether this signature of population growth testifies for independent events of expansion in the different populations here analyzed or a common and major event of drastic, recent population growth (e.g. the Bantu expansion) should be the object of future studies.

Our data also support the notion that both Europeans and East-Asians descended from the same diffusion event expanding out of Africa. Indeed, we show that the most probable model involved an out-of-Africa event occurring ∼60,000 (47,000–85,000) years ago, followed by a much later diversification of non-African populations ∼23,000 (17,000–35,000) years ago. Such a late diversification of Eurasian populations after the out-of-Africa exodus suggests the existence of an ancestral population (stationary or expanding) located somewhere central in the Eurasian continent at the basis of the present-day Europeans and East Asians. Several studies, mostly based on uniparentally inherited markers, have shown that Central Asian populations harbor genetic features that are intermediate between Europeans and East-Asians [66]–[68]. In addition, our estimated time of the split of Eurasian populations of ∼23,000 years ago appears to be slightly more recent than the archaeological and fossil records of Aurignacian technologies and skeletal remains of diagnostically modern humans in Europe (Cro-Magnon) dating to around 30,000–40,000 years ago [69]–[71]. This points to a further layer of complexity of the mode and rhythm of the old-world colonization, which may have involved multiple migration waves associated with several bottlenecks of different intensities starting at different ages from the ancestral Eurasian population pool. Resequencing studies of unlinked, noncoding, multiple loci in ethnologically well-defined populations from Central Asia are needed to address this question in the context of Eurasian prehistory. Finally, this study, together with a recent analysis focused on Central African populations [72], allowed us to co-estimate levels of divergence and gene flow in humans, by using an ABC framework. Our analyses have estimated a non-negligible gene flow between continental populations, which is equivalent to a symmetric constant migration rate of ∼10^−5^ per generation. Theoretical simulation studies should help to discern whether this observation corresponds to a genuine average between-continent migration rate over time or reflects instead varying temporal intensities of migration rates (symmetric or asymmetric).

An additional improvement of our analytical approach is determining the accuracy of parameter co-estimation under ABC. Our analyses allowed us to identify the most accurate set of statistics to be used for the estimation of a given parameter and indicated that no general rule can be proposed to select a specific combination of summary statistics – the set of summary statistics providing the best accuracy varies depending on the parameter to be estimated. We also showed that our parameter estimations are robust both to the shape of the prior distributions used and to the choice of the model of human dispersals out of Africa. More importantly, our accuracy testing procedure identified two parameters that are probably unreliable: the present-day African effective population size, ***N_A_***, which exhibited high bias (*B*), standard error (*SE*) and root of mean square error (*RMSE*) (Table 3), and the replacement rate, ***δ***, which was sensitive to the shape of the prior distributions. It is worth noting that, despite the accuracy statistics pointed to low biases in the estimation of the growth rate, ***α_A_***, of the African expansion, this parameter presented a posterior distribution that largely overlapped its prior distribution.

In conclusion, our study provides a refined model of the historical and demographic parameters occurring in the last 100,000 years. Formulating a model of human demography based on neutral, or quasi-neutral, polymorphisms has implications that go beyond understanding human evolution. It provides background expectations about population genetic variation, increasing our understanding about the population frequency of disease-causing alleles, facilitating the estimation of recombination rates from patterns of linkage disequilibrium, and allowing robust identification of regions of the genome targeted by natural selection [2], [13], [14]. By providing the posterior distributions of the demographic parameters, rather than point estimates, our work gives access to genetic variability from non-standard population genetic models and estimates of uncertainty. Indeed, neglecting this latter aspect of variability by performing simulations with point estimates (such as maximum likelihood) used as true parameter values could also bias the detection of natural selection. Our data, together with other studies based on noncoding resequencing data from other human populations [38], [40], [43], [44], contribute to a common consensual model of recent human evolution that can be used in the context of disease-mapping studies and inferences of natural selection. However, this general picture may still be overly simple because current genetic data are still limited and do not permit differentiation of simple models from more complex realistic models involving, for example, varying intensities of migration rates between populations over time, long-range expansions, or sexually-asymmetric mating patterns. Additional sequence-based data from large, ethnologically well-defined populations are clearly needed to obtain a more refined and unbiased picture of the demographic history of human populations. In this context, the 1000 Genomes Project, which involves the sequencing of entire genomes of at least a thousand people from around the world, will contribute with massive amounts of data and will provide a more precise idea of different demographic events of recent human history. In parallel, theoretical work on more sophisticated models of human demography and improved methods of data analyses are undoubtedly required.

## Materials and Methods

### DNA Samples

Sequence variation was surveyed in DNA samples from 213 healthy donors. The panel included 118 sub-Saharan African individuals represented by 5 agriculturalist populations, including Yoruba from Nigeria (N = 31), Ngumba from Cameroon (N = 16), Akele from Gabon (N = 16), Chagga from Tanzania (N = 32), and Mozambicans (N = 23), 47 European individuals represented by Danes (N = 23) and Chuvash from Russia (N = 24), and 48 East-Asian individuals represented by Han Chinese (N = 24) and Japanese (N = 24). Informed consent (written) was obtained from each anonymous, voluntary participant. In specific cases where participants were not literate enough to read and sign a form, oral consent was obtained for this ethnographic study. All these procedures and study materials were specifically approved by the Institut Pasteur Institutional Review Board (n° RBM 2008.06).

### Resequencing Data

We selected 20 autosomal regions (Table S1) that met criteria determined by the need for genetic variation evolving under selective neutrality and therefore influenced by demography alone. Regions were thus selected (i) to be independent from each other, (ii) to reside at least 200 kb apart from any known or predicted gene or spliced expressed sequence tag (EST) (mean distance of 760 kb and 390 kb from genes and spliced ESTs, respectively, as determined by inspection of the hg18 UCSC genome assembly), (iii) not to be in LD with any known or predicted gene or spliced EST (as determined by inspection of LD levels observed in the four HapMap populations, release 16), and (iv) to have a region of homology in the chimpanzee genome (November, 2003, release).

All 20 autosomal regions were sequenced with two different primers, for a total sequence length of ∼27 kb per individual (mean sequence length per region of ∼1.33 kb). PCR and sequencing primers and protocols are available upon request. All sequencing reactions were run on automated capillary sequencers (ABI3130 and ABI3730). Sequence alignment and SNP detection were performed using Genalys v.3.3b [73]. In addition, all ABI base-calling sequences were visually inspected by two independent investigators. All singletons were confirmed by re-amplification and resequencing. No false singleton was observed. Less than 0.1% of genotypes were considered as missing data. All the 20 genomic regions were found to be polymorphic over the 213 resequenced individuals, as expected given the number of polymorphic sites (*S*) under the neutral mutation model [74]; E(*S*) = a_1_4*N_e_μ* = 7.9, where a_1_ is the sum of 1/i, with i varying from 1 to n-1 (*n* being the sample size of 213 individuals), *N_e_* is the effective population size of the population (*N_e_* = 10,000 in humans) and *μ* the mutation rate per generation per DNA sequence under investigation (i.e. the product of the mutation rate per generation per site, which equals to 2.5×10^−8^ [39], [40], and the length of DNA sequence, which equals to 1330 bp in average).

### Summary Statistics

Haplotype reconstruction was performed using the Bayesian method implemented in PHASE v2.1 [75], [76]. All samples were merged to take advantage of the large sample size (213 individuals). Indeed, the geographical structure of populations does not affect the average accuracy of the PHASE algorithm [76]. The number of iterations, the thinning interval, and the burn-in length were set to 1000, 100, and 1000 respectively. Each iteration consists of performing “thinning interval” steps through the Markov chain, and each step updates each individual once. Five independent Markov chains were run, each with a different seed, and we systematically chose the phase reconstruction with the highest posterior probability.

We computed the observed and expected minor allele frequency (MAF) spectra using DnaSP software [77]. The expected MAF spectra were computed assuming continental human populations of constant sizes and using individual **θ** (**θ** = 4Nμ) estimated from the sub-Saharan African, the European, and the East-Asian samples. The deviations between observed and expected proportions of singletons were tested using a χ^2^ test, with 1 degree of freedom, after summarizing MAF into two classes (singletons and non-singletons). To compute the observed derived allele frequency (DAF) spectra, we retrieved for each identified SNP the ancestral allelic state. To this end, we aligned the human sequence containing a given SNP with genomes of other primates (*Pan troglodytes*, *Pongo pygmaeus*, *Macacca mullata*; UCSC database) and deduced by parsimony the ancestral state of the SNP. The expected DAF spectra were obtained by simulating continental samples assuming populations of constant size and following the simulation procedure detailed below. The deviations between observed and expected proportions of fixed derived alleles were tested using a χ^2^ test, with 1 degree of freedom, after summarizing DAF into two classes (fixed derived alleles and non-fixed derived alleles).

We computed summary statistics using a modified version of ARLEQUIN v3 [78]. For each genomic region, we computed population differentiation indices, including global and pairwise *F*_ST_ [79] based on haplotype frequencies. To accommodate different aspects of the resequencing dataset, we also computed for each genomic region the number of haplotypes, *K*, the number of polymorphisms, *S*, the nucleotide diversity, *π*, Tajima's *D* [74], Fu's *Fs* [80], Fu and Li's F* [81], and Fay and Wu's *H* [82] statistics. We computed these summary statistics for each continental sample separately and also merging all samples together. Means and standard deviations of these statistics over the 20 autosomal regions were also computed to combine information from multiple loci.

### Simulations of Genetic Data

Simulations were performed using a generation per generation coalescent-based algorithm, implemented in SIMCOAL v2 [83]. Simulated summary statistics were computed using a modified version of ARLEQUIN v3 [78]. The general algorithm to perform simulations is: 1) draw parameters from specified random distributions, 2) call SIMCOAL v2 to simulate datasets according to specified parameters, 3) call modified ARLEQUIN v3 to compute all required summary statistics for the simulated dataset, and 4) go back to 1) for the next simulation. This procedure was computationally intensive, and was performed using a cluster of 10 bi-processor (64 bits, 1.8 GHz, 2 GB RAM) computers running on the Linux operating system. Using this algorithm, we simulated DNA sequences of 1,400 bp each. The mutation and the recombination rates of each region were drawn from gamma distributions in accordance with previous studies [39], [40]. As to the mutation rate, we used a finite site mutation model with a per generation per site mutation rate, gamma distributed with a mean of ∼2.5×10^−8^ and a 95% confidence interval of 1.47×10^−8^ to 4.03×10^−8^. As to the recombination rate, we considered between two adjacent base pairs, a per generation recombination rate, gamma distributed with a mean ∼10^−8^ and a 95% confidence interval of 0.48×10^−8^ to 1.43×10^−8^.

### Simulations of the Constant Population Size Model

To test for deviations of the observed derived allele frequency (DAF) spectra and summary statistics (global and pairwise *F*_ST_, *K*, *S*, *π*, Tajima's *D*, Fu's *Fs*, Fu and Li's F* and Fay and Wu's *H*) from the null assumption of constant population size, we performed 10^5^ simulations of 20 independent regions drawing for each simulation the mutation rate and effective population sizes from gamma distributions described above. Because it is difficult to accurately estimate the recombination rate, we tested three different procedures to model it. First, we neglected intra-region recombination; this option is justified because we only observed ∼0.5% of recombinant haplotypes in the 20 autosomal genomic regions using the four-gamete test (data not shown). Second, we assumed a per generation intra-region recombination rate between adjacent base pairs that was gamma-distributed with a mean of ∼10^−8^ (95% confidence interval of 0.48×10^−8^ to 1.43×10^−8^) [39], [40]. Third, we assumed a per generation intra-region recombination rate fixed 10 times higher than expected in humans (i.e., equal to 10^−7^ between adjacent base pairs). For each configuration, 10^5^ simulations of three independent populations were performed, with sample sizes corresponding to sub-Saharan African, European, and East-Asian samples (118, 47, and 48 individuals, respectively). *P*-values for deviations from the constant population size model were computed by counting the number of simulated summary statistics with values higher or lower than the observed summary statistics.

### Simulations of Demographic Histories

To explore the space of demographic parameters we aimed to investigate, we treated them as continuous random variables with prior distributions, rather than performing simulations over grids of discrete parameter values [9], [40]. All demographic events were chosen to be uniformly distributed (i.e. flat prior distributions) except the effective size of populations. Under equilibrium assumptions, the human effective population size has been estimated at ∼10,000 individuals on the basis of human-chimp divergence and intra-species LD levels [4], [84]. To both give population size a degree of freedom and to match with a consensus estimate of human populations, we defined a gamma prior distribution with a mean of ∼10,000 individuals and a 95% confidence interval of 3,000 to 21,000 individuals [39], [40]. Note that when simulating population expansions, we excluded simulations with values of expansion parameters resulting in present-day effective population sizes exceeding 1 billion individuals.

### General Statistical Procedures to Co-estimate Historical and Demographic Parameters

To explore and co-estimate a range of historical and demographic parameters, we adopted a two-step procedure as previously described [72]. In the first step, we evaluated multiple models of human evolution using a best-fit approach performed in order to decrease the number of models and the parameter space to be efficiently explored in the second step. In this second step, we co-estimated parameters of interest using a Bayesian approach, which made use of model and parameter priors best fitted in the first step. We finally systematically checked for the accuracy of the parameter co-estimations.

#### First step: the best-fit approach

We adopted the same flexible statistical framework implemented in [72] and inspired by previous methods [47], [49]. For both the adjustment of the global evolutionary scenario and the demographic regimes of each continental group, we generated for each model 10^5^ simulated datasets of 20 unlinked DNA sequences (∼1,400 bp each) in 118 sub-Saharan African, 47 European, and 48 East-Asian individuals. The simulated model that best fitted our autosomal data was defined as that giving the highest proportion of small distances (*ψ_ξ_*) between the simulated and observed summary statistics, S' and S. These distances were measured by calculating the normalized metric *D*(S',S) [38], and *D*(S',S) was considered to be small when lower than a *ξ* value, e.g. *ψ_ξ_* = 0.1 means that 10% of all distances are smaller that *ξ*. To include multi-locus information in calculating these metrics, we used the mean, for each summary statistics, computed over the 20 autosomal non-coding regions. To assess whether a given model fitted the empirical data significantly better than another model, we resampled 100 times 10,000 simulations of each model. We next calculated the *ψ_ξ_* for each resampling set. For each model, we computed the mean *ψ_ξ_* over the 100 resampling sets. We tested for significant differences between the mean *ψ_ξ_* of the different models, using a Student's t-test followed by a Bonferroni correction for multiple testing (multiple pairwise comparisons). Finally, classes of models exhibiting the highest mean *ψ_ξ_*, and that were statistically indistinguishable, were all retained to construct the best-fit model. We also tested the extent to which the choice of the model based on the highest *ψ_ξ_* can provide a false model (e. g. over fitting due to high number of parameters, etc.). To this effect, we simulated 100 datasets under each tested model and used them as if they were empirical data. For example, let us consider 1 simulated pseudodataset generated under model M_1_, and an alternative model M_2_ to be tested. We calculated, for this simulated pseudodataset, *ψ_ξ_* for M_1_ and *ψ_ξ_* for M_2_. If *ψ_ξ_* for M_1_>*ψ_ξ_* for M_2_, then the best-fit model (highest *ψ_ξ_*) corresponds to the “correct” model (M_1_), or else (*ψ_ξ_* for M_1_<*ψ_ξ_* for M_2_), the highest *ψ_ξ_* corresponds to a “wrong” alternative model (here M_2_). Therefore among the 200 simulated pseudodatasets (100 simulated under M_1_ and 100 simulated under M_2_), we counted the number of times where the highest *ψ_ξ_* was obtained for the correct simulated model (M_1_ or M_2_ depending on the pseudodataset used). This count divided by 200 (the total number of simulated pseudodatasets) was used as a proxy of the probability to obtain the “true model” taking into account the high variability of datasets that can be obtained under a given demographic scenario. We used this approach to perform pairwise comparisons between the best-fit model (highest *ψ_ξ_* obtained using our true empirical dataset) against many other alternative models.

#### Second step: Co-estimation of parameters by Approximate Bayesian Computation

The first step was used to decrease the model and parameter space to be subsequently explored in the Approximate Bayesian Computation (ABC) [46], [85] co-estimation of historical and demographic parameters. Given the complexity of the historical and demographic models we aimed to explore, we sought to overcome the problem of unknown likelihood functions [38], [72] by using the ABC setting. ABC approaches bypass the computational difficulties of using explicit likelihood functions by simulating data from a coalescent model, and thus provide high degree of freedom in the choice of demographic models to be tested. These methods rely on the simulation of large numbers of datasets using parameter values sampled from prior distributions, i. e. the parameter ranges of variation determined by means of the best-fit approach used in the first step of this study. A set of summary statistics is then calculated for each simulated sample, and each set of simulated statistics is then compared with the values observed in the empirical data using the normalized metrics *D*(S',S), with S' the simulated and S the empirical summary statistics [38]. Similarly to the first step, we used the mean of summary statistics over the 20 autosomal non-coding regions. Parameter values generating summary statistics similar enough to those of the empirical data were retained, i.e. the 5,000 simulations with the smallest D(S',S). Posterior distributions of the parameters were obtained with a locally weighted multivariate regression [38], [46]. We generated 10^6^ simulated datasets of 20 unlinked DNA sequences (∼1,400 bp each) in 118 sub-Saharan African, 47 European, and 48 East-Asian individuals using the model that best fit our data, i. e. the combination of ranges of parameters determined in the first step of this study.

#### Tests for the accuracy and validation of parameter estimations

There is no general rule in the ABC procedure to choose which combination of summary statistics (Table S10) outperforms the others, because no combination would be sufficient to account for all aspects of the data. For example, the use of summary statistics that are not correlated with the unknown parameter could potentially introduce noise and alter the estimation accuracy. Furthermore, different point estimators (i.e. the mean, the median and the mode of distribution) can be computed from posterior distributions, and there is no satisfactory rule to determine which estimator outperforms the others. We therefore systematically tested for different combinations of summary statistics and different point estimators, by simulating 100 datasets under the best-fit model. These datasets were considered as “pseudo-empirical” datasets. Indeed, we re-estimated the underlying known parameters for each of these 100 “pseudo-empirical” datasets with exactly the same approach used for the ABC estimation performed with the empirical dataset (i. e. the 10^6^ simulations of the best-fit model). We then compared the re-estimated values of parameters with their known values. We used different accuracy indices: the relative bias (difference between expected and estimated values expressed as a percent of the known value), the relative standard error (the standard error expressed as a percent of the known value), and the relative root mean square error (*RMSE*) (the mean square error expressed as a percent of the known value). The *RMSE* statistic is commonly used to determine which estimation is the most accurate, because the method with the smallest *RMSE* should provide estimates with the lowest combination of bias and variance. For each parameter, we therefore retained the point estimate and the combination of summary statistics yielding the lowest root of mean square error, *RMSE*, to provide the most reliable estimation.

Finally, we evaluated the sensitivity of our co-estimations (2^nd^ step) to the prior distributions calibrated using our best-fit approach (1^st^ step). Indeed, in Bayesian settings, the choice of priors is a crucial but difficult question to address. In principle, changes in the prior definition of parameters should not alter the posterior estimations. We therefore performed simulations using modified prior distributions of the selected parameter, keeping other prior distributions unchanged to avoid strong inflation of the global parameter space. Indeed, this inflation could disturb estimation when using limited numbers of simulated datasets. We modified priors by simulating extended ranges and/or modified shapes of prior distributions (determined in 1^st^ step, see above), and we used our empirical data to re-estimate each parameter with the newly defined prior distributions. Because performing all these tests is computationally costly, we decreased the number of simulations (10^5^ rather than the 10^6^ simulations initially performed to estimate parameters).

### Web Resources

Arlequin v.3.11, http://cmpg.unibe.ch/software/arlequin3/

Chimpanzee Genome Resources, http://www.ncbi.nlm.nih.gov/genome/guide/chimp/

DnaSP v. 4.1, http://www.ub.es/dnasp/

GenBank, http://www.ncbi.nlm.nih.gov/Genbank/ [accession numbers GU462347 – GU470440])

HapMap database, http://www.hapmap.org/index.html.en

PHASE v2.1.1, http://www.stat.washington.edu/stephens/software.html

SIMCOAL v. 2.0, http://cmpg.unibe.ch/software/simcoal2/

UCSC database, http://genome.ucsc.edu/

## Supporting Information

Figure S1 Effects of bottleneck intensity on the number of haplotypes, the number of polymorphic sites and Fay and Wu's H statistics.
(0.07 MB DOC)

Figure S2 Schemes of the simulated demographic models.
(0.07 MB DOC)

Figure S3 The mimicking effects of migrations and expansions.
(0.03 MB DOC)

Figure S4 New approximate posterior distributions after altering the prior distributions.
(0.05 MB DOC)

Figure S5 Approximate posterior distributions computed using the four alternative dispersal models out of Africa.
(0.95 MB DOC)

Table S1 Genomic features of the 20 independent autosomal non-coding regions sequenced in this study.
(0.09 MB DOC)

Table S2 Summary statistics and neutrality tests of the 20 genomic regions considering various recombination rates.
(0.20 MB DOC)

Table S3 Matrix of pairwise FST computed between ethnic groups.
(0.04 MB DOC)

Table S4 Description of the prior distributions of historical and demographic parameters simulated.
(0.14 MB DOC)

Table S5 Testing the accuracy of ABC estimations using different sets of summary statistics.
(0.06 MB DOC)

Table S6 ABC estimations of parameters using different sets of summary statistics.
(0.05 MB DOC)

Table S7 Testing the influence of prior distributions on parameter estimations.
(0.04 MB DOC)

Table S8 Testing the influence of prior distributions for some parameters on the estimation of other parameters.
(0.05 MB DOC)

Table S9 Testing the influence of the models of human dispersals out of Africa used, on parameter estimations.
(0.04 MB DOC)

Table S10 List of summary statistics used.
(0.05 MB DOC)
