# Supplementary material for: Formulating a Historical and Demographic Model of Recent Human Evolution Based on Resequencing Data from Noncoding Regions
Source: PLoS One. 2010 Apr 22;5(4):e10284. doi: 10.1371/journal.pone.0010284 (PMC2858654; doi:10.1371/journal.pone.0010284)

**Fig. S1** Effects of bottleneck intensity on the number of haplotypes, the number of polymorphic sites and Fay and Wu’s *H* statistics

We simulated 20 genomic independent regions of ~1,400 bp each, which followed a finite site mutation model with a per generation per site mutation parameter gamma distributed with mean ~2.5x10^-8^ with 95% confidence intervals ranged from 1.47x10^-8^ to 4.03x10^-8^. We simulated three sample sizes (with no migration) corresponding to sub-Saharan African (118 individuals), European (47 individuals) and East-Asian (48 individuals) populations. We simulated several bottleneck intensities, defined as the ratio between the effective population sizes before and after the bottleneck. We performed 10^5^ computer simulations of a population with constant effective size, gamma distributed with average equal to N=10,000 individuals, who experienced a size reduction 100 generations ago. The intensities of the simulated bottlenecks were of 1 (no bottleneck), 10, 20, 50 and 100. Reduction of the **(A)** mean and (**B)** standard deviations of the numbers of haplotypes (K) and polymorphic sites (S), computed over 20 simulated regions with respect to various simulated bottleneck intensities. (**C)** Reduction of the mean of the Fay and Wu’s *H* statistics computed over the 20 simulated regions with respect to various simulated bottleneck intensities. (**D)** Bi-plot of the per region numbers of haplotypes and polymorphic sites simulated assuming constant population sizes (grey) and a bottleneck intensity equal to 50 (black).

**D**

**B**

**C**

**A**


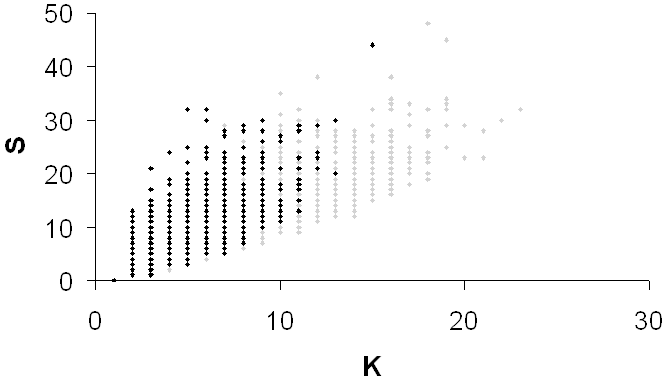

Supplement: Figure S1 — Effects of bottleneck intensity on the number of haplotypes, the number of polymorphic sites and Fay and Wu's H statistics. (0.07 MB DOC) [file pone.0010284.s001.doc]
