# Supplementary material for: Formulating a Historical and Demographic Model of Recent Human Evolution Based on Resequencing Data from Noncoding Regions
Source: PLoS One. 2010 Apr 22;5(4):e10284. doi: 10.1371/journal.pone.0010284 (PMC2858654; doi:10.1371/journal.pone.0010284)
Supplement: Figure S2 — Schemes of the simulated demographic models. (0.07 MB DOC) [file pone.0010284.s002.doc]

**Fig. S2** Schemes of the simulated demographic models

**(A-D)** Models tested: **(A)** No replacement of ancestral European and Asian populations by African modern humans (δ =0) to **(D)** complete replacement of ancestral European and Asian populations (δ =1). The replacement rate parameter δ allowed us to simulate various intensities of replacement: **(B)** δ <0.5 and **(C)** RAOI δ >0.5. A replacement rate lower than 0.5 means that less than 50% (dark grey) of current lineages have been brought by the Out-of-Africa exodus of modern humans. On the contrary, a replacement rate higher than 0.5 means that more than 50% (light grey) of current lineages has been brought by the Out-of-Africa exodus of modern humans. The distributions of parameters used to simulate these models are specified in Table S4.

**AS**

**EU**

**AF**

**C**

**D**

**AS**

**EU**

**AF**

**AS**

**EU**

**AF**

**A**

**AS**

**EU**

**AF**

**B**

**δ**

**δ**

**δ**

**δ**

**δ**

**δ**

**δ**

**δ**

**δ=0**

**0≤δ≤0.5**

**0.5≤δ≤1**

**δ=1**
