# Supplementary material for: Formulating a Historical and Demographic Model of Recent Human Evolution Based on Resequencing Data from Noncoding Regions
Source: PLoS One. 2010 Apr 22;5(4):e10284. doi: 10.1371/journal.pone.0010284 (PMC2858654; doi:10.1371/journal.pone.0010284)
Supplement: Figure S3 — The mimicking effects of migrations and expansions. (0.03 MB DOC) [file pone.0010284.s003.doc]

**Fig. S3** The mimicking effects of migrations and expansions

We simulated one region of ~1,400 bp, which followed a finite site mutation models with per generation per site mutation parameter gamma distributed with mean ~2.5x10^-8^, with 95% confidence intervals ranging from 1.47x10^-8^ to 4.03x10^-8^. Between two adjacent base pairs, we considered a per generation recombination rate parameter gamma distributed with mean ~10^-8^, with 95% confidence intervals ranging from 0.48x10^-8^ to 1.43x10^-8^. We simulated 3 populations diverging ~75,000 years ago, a divergence date mimicking the expected divergence time in humans (*F*_ST_ in the right Y axis). The first population, which is represented as black diamonds, simulates a population with a current effective size N=1,000,000 that experienced a 100-fold instantaneous expansion 2,500 generations in the past (Tajima’s *D* = -1.8, represented in the left Y axis). The second population, which is represented as black triangles, simulates a population with current effective size N=10,000 that keep constant over time. The third population, which is represented as white triangles, simulates a population with current effective size N=1000 that experienced a 10-fold bottleneck 100 generations in the past. Immediately after divergence, the expanding population (black diamonds) sent migrants to the others. We simulated several migration rate intensities (X axis) which correspond to the following number of migrants per generation kept constant over time: 0, 3, 7.5, 30, 75, 150 and 500. This divergence/migration scheme generated *F*_ST_ (white circles coupled with the right Y axis) that are generally realistic in humans. While the Tajima’s *D* is unchanged in the expanding population (no incoming migrants in this population), the Tajima’s *D* largely decreased with the intensity of the proportion of incoming migrants (X axis) removing the signal of bottleneck in the third population and mimicking an expansion signal in the second (constant) population. Interestingly, even a moderate decrease of *F*_ST_ due to migration (*F*_ST_ value decreasing to 0.08, forth white circle dot) results in a strongly false expansion signal (Tajima’s *D* decreasing to -0.41, fourth black triangle dot) while the bottleneck signal is considerably erased (Tajima’s *D* decreasing to -0.13, fourth white triangle dot).
