# Supplementary material for: Formulating a Historical and Demographic Model of Recent Human Evolution Based on Resequencing Data from Noncoding Regions
Source: PLoS One. 2010 Apr 22;5(4):e10284. doi: 10.1371/journal.pone.0010284 (PMC2858654; doi:10.1371/journal.pone.0010284)
Supplement: Figure S4 — New approximate posterior distributions after altering the prior distributions. (0.05 MB DOC) [file pone.0010284.s004.doc]

**Fig. S4** New approximate posterior distributions after altering the prior distributions

This figure shows the prior (black curves) and posterior distributions (grey bars) of estimations given in Table 3 and Figure 5. The posterior distributions obtained after extending the prior distributions (red curves) are represented as orange bars. For all estimations, posterior distributions are similar to those previously obtained. The only exception concerns the replacement rate δ, where the two new posterior distribution (obtained using the two new enlarged prior ranges) largely exceeded the confidence interval (0.99 - 0.9997, Table 3) from the previously estimated posterior distribution.

***t_A_***

Time (number of years / 10^3^)

***α_A_***

Rate (per generation)

Number of individuals (10^3^)

***N’***

***T_OoA_***

***δ***

Time (number of years / 10^3^)

Replacement rate

***m***

Modern human migration rate between continents
