# Supplementary material for: Formulating a Historical and Demographic Model of Recent Human Evolution Based on Resequencing Data from Noncoding Regions
Source: PLoS One. 2010 Apr 22;5(4):e10284. doi: 10.1371/journal.pone.0010284 (PMC2858654; doi:10.1371/journal.pone.0010284)

**Fig. S5** Approximate posterior distributions computed using the four alternative dispersal models out of Africa

The symbol of each parameter is given in Table 2. The 10^5^ simulations were performed using the four models of dispersal after the Out-of-Africa exodus (Figures 4A-D). Each simulation was performed by giving the same probability to each model. For each histogram, black lines represent the prior distributions and grey bars represent the posterior distributions. The histogram of the models shows the posterior probability of each of the four alternative assumptions (model A corresponds to Figure 4A, model B to Figure 4B, model C to Figure 4C and model D to Figure 4D). The parameter of the time of the single dispersal out of Africa (***T_OoA_***, boxed parameter) was estimated considering only models A and B, because the remaining two models consider multiple dispersals out of Africa (i.e. the time of the Out-of-Africa exodus parameter in models C and D is divided into two different parameters). The posterior distributions of the parameters where the estimations were not validated with the accuracy evaluation procedure are not presented (***N_A_*** and ***δ***).


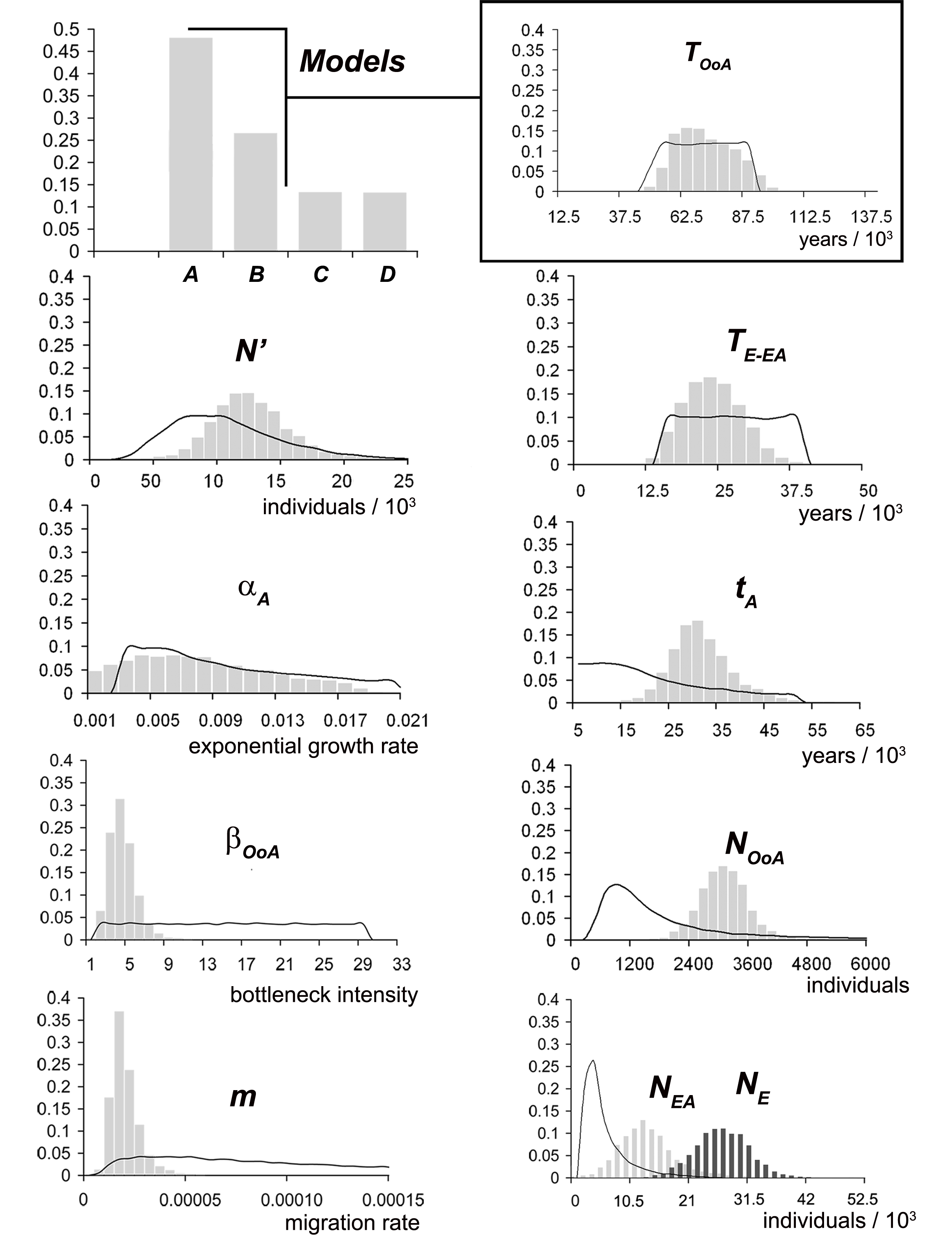

Supplement: Figure S5 — Approximate posterior distributions computed using the four alternative dispersal models out of Africa. (0.95 MB DOC) [file pone.0010284.s005.doc]
