# Supplementary material for: Formulating a Historical and Demographic Model of Recent Human Evolution Based on Resequencing Data from Noncoding Regions
Source: PLoS One. 2010 Apr 22;5(4):e10284. doi: 10.1371/journal.pone.0010284 (PMC2858654; doi:10.1371/journal.pone.0010284)
Supplement: Table S1 — Genomic features of the 20 independent autosomal non-coding regions sequenced in this study. (0.09 MB DOC) [file pone.0010284.s006.doc]

**Table S1.** Genomic features of the 20 independent autosomal non-coding regions sequenced in this study

| **Region** | **Chr^a^** | **Length (bp)** | **Position** | **5'-closest EST** | **Distance (bp)** | **3'-closest EST** | **Distance (bp)** | **5'-closest gene** | **Distance (bp)** | **3'-closest gene** | **Distance (bp)** | **Segregating sites^b^** |
| --- | --- | --- | --- | --- | --- | --- | --- | --- | --- | --- | --- | --- |
|  |  |  |  |  |  |  |  |  |  |  |  |  |
| R04 | 1p | 1328 | 106787624-106788951 | *AA954094* | 358983 | *BU854483* | 373433 | *BC043293* | 824544 | *PRMT6* | 611981 | 16 |
| R05 | 2p | 1271 | 76163330-76164600 | *BF967228* | 363160 | *DB302076* | 664592 | *c2orf3* | 371500 | *UNQ3075* | 664592 | 18 |
| R06 | 2q | 1300 | 117090864-117092163 | *BC030832* | 772458 | *DA742384* | 771905 | *DPP10* | 772458 | *DQ571524* | 405462 | 21 |
| R07 | 3p | 1373 | 20665087-20666459 | *AK026452* | 258029 | *CF618845* | 255538 | *SGOL1* | 462400 | *HPX-42* | 755763 | 22 |
| R08 | 3q | 1371 | 146671295-146672665 | *AI693058* | 460613 | *CA453629* | 348970 | *C3orf58* | 1477404 | *DQ595575* | 352302 | 9 |
| R09 | 4p | 1256 | 29547438-29548693 | *DB451145* | 269619 | *BG216723* | 727133 | *FLJ45721* | 2718314 | *PCDH7* | 782442 | 16 |
| R10 | 4q | 1326 | 179693433-179694758 | *BC033326* | 544535 | *BC043428* | 438222 | *BC033326* | 544535 | *AF088005* | 2527479 | 22 |
| R14 | 6q | 1300 | 91757485-91758784 | *BE044076* | 400143 | *BC037927* | 636691 | *MAP3K7* | 403857 | *BC037927* | 636691 | 12 |
| R16 | 7p | 1401 | 13052349-13053749 | *BU664973* | 259878 | *DA181168* | 53792 | *ARL4A* | 355266 | *ETV1* | 843632 | 22 |
| R17 | 7q | 1350 | 118479068-118480417 | *AA018891* | 564369 | *CB338058* | 566302 | *ANKRD7* | 809087 | *KCND2* | 1220541 | 19 |
| R18 | 8p | 1256 | 5139032-5140287 | *DB446481* | 167686 | *BC040995* | 210712 | *CSMD1* | 299296 | *CR623475* | 1108198 | 33 |
| R20 | 8q | 1282 | 137174739-137176020 | *DB092925* | 405774 | *DA593750* | 435281 | *KHDRBS3* | 445709 | *C8ORFK32* | 2035428 | 15 |
| R21 | 9p | 1390 | 11567765-11569154 | *DA212144* | 223691 | *DB098556* | 39340 | *PTPRD* | 3263519 | *TYRP1* | 1131103 | 23 |
| R25 | 11q | 1340 | 80750645-80751984 | *AK001959* | 525369 | *BC041900* | 516560 | *AF009227* | 2146040 | *BC041900* | 516560 | 16 |
| R26 | 11q | 1330 | 96959343-96960672 | *BF575990* | 292539 | *BG182718* | 358300 | *JRKL* | 1192968 | *CNTN5* | 1436409 | 17 |
| R30 | 14q | 1345 | 82997591-82998935 | *DB451719* | 418416 | *AI217957* | 444034 | *BC029835* | 1838433 | *BX248253* | 2062295 | 16 |
| R32 | 15q | 1301 | 84958820-84960120 | *CD359326* | 297622 | *AK096897* | 300270 | *AGBL1* | 318370 | *TMEM83* | 961044 | 12 |
| R33 | 16q | 1350 | 58084866-58086215 | *AK057513* | 384487 | *AA782991* | 215183 | *GOT2* | 759119 | *CDH8* | 2158521 | 24 |
| R36 | 18q | 1200 | 26002122-26003321 | *BG461864* | 619942 | *DA340389* | 543921 | *CDH2* | 1990933 | *CR600534* | 820651 | 12 |
| R42 | 20q | 1347 | 53069630-53070976 | *BC008992* | 368795 | *DB033847* | 271799 | *DOK5* | 368513 | *CBLN4* | 934927 | 16 |
|  |  |  |  |  |  |  |  |  |  |  |  |  |
| **Mean** |  | 1321 |  |  | 397805 |  | 408599 |  | 1068113 |  | 1098301 | 18.05 |
|  |  |  |  |  |  |  |  |  |  |  |  |  |

^a^ Chromosomal region

^b^ Number of segregating sites computed across the 213 individuals

Note. ESTs refer here to mRNAs or spliced ESTs, and genes refer to UCSC gene predictions or RefSeq genes.
