# Supplementary material for: Formulating a Historical and Demographic Model of Recent Human Evolution Based on Resequencing Data from Noncoding Regions
Source: PLoS One. 2010 Apr 22;5(4):e10284. doi: 10.1371/journal.pone.0010284 (PMC2858654; doi:10.1371/journal.pone.0010284)
Supplement: Table S2 — Summary statistics and neutrality tests of the 20 genomic regions considering various recombination rates. (0.20 MB DOC) [file pone.0010284.s007.doc]

**Table S2.** Summary statistics and neutrality tests of the 20 genomic regions considering various recombination rates

|  | **r^a^** |  | ***K*** | ***S*** | ***π%*** | ***D*** | ***Fs*** | ***F**** | ***H*** |  |  |  | ***F*_ST_** | ***A-E F*_ST_** | ***A-A F*_ST_** | ***E-A F*_ST_** |
| --- | --- | --- | --- | --- | --- | --- | --- | --- | --- | --- | --- | --- | --- | --- | --- | --- |
|  |  |  |  |  |  |  |  |  |  |  |  |  |  |  |  |  |
| ***Sub-Saharan Africans*** | |  |  |  |  | ***mean*** |  |  |  |  |  |  |  |  | ***mean*** |  |
|  |  | **Obs** | **15.15** | **14.20** | **0.12** | **-0.85** | **-5.75** | **-1.75** | **-0.25** |  |  | **Obs** | **0.127** | **0.122** | **0.146** | **0.049** |
|  | r=0 | Exp | 7.61 | 8.48 | 0.11 | -0.03 | -0.26 | -0.03 | 0.03 |  | R=0 | Exp | 0.108 | 0.116 | 0.116 | 0.049 |
|  |  | *P* | ***0.0000*** | ***0.0000*** | *0.3260* | ***0.0000*** | ***0.0000*** | ***0.0000*** | *0.0948* |  |  | *P* | *0.1545* | *0.4033* | *0.1010* | *0.4401* |
|  | r=10^-8^ | Exp | 7.93 | 8.52 | 0.11 | -0.04 | -0.49 | -0.03 | 0.02 |  | R=10^-8^ | Exp | 0.109 | 0.117 | 0.117 | 0.051 |
|  |  | *P* | ***0.0000*** | ***0.0000*** | *0.3149* | ***0.0000*** | ***0.0000*** | ***0.0000*** | *0.1015* |  |  | *P* | *0.168* | *0.423* | *0.102* | *0.481* |
|  | r=10^-7^ | Exp | 9.84 | 8.43 | 0.11 | -0.02 | -1.74 | 0.00 | 0.01 |  | R=10^-7^ | Exp | 0.108 | 0.116 | 0.117 | 0.050 |
|  |  | *P* | ***0.0006*** | ***0.0002*** | *0.2953* | ***0.0000*** | ***0.0000*** | ***0.0000*** | *0.0954* |  |  | *P* | *0.1414* | *0.3971* | *0.0929* | *0.4563* |
|  |  |  |  |  |  | ***SE*** |  |  |  |  |  |  |  |  | ***SE*** |  |
|  |  | **Obs** | **5.04** | **5.35** | **0.06** | **0.63** | **3.94** | **1.30** | **0.76** |  |  | **Obs** | **0.096** | **0.091** | **0.123** | **0.044** |
|  | r=0 | Exp | 3.69 | 5.53 | 0.09 | 0.94 | 2.99 | 0.99 | 0.87 |  | R=0 | Exp | 0.092 | 0.109 | 0.109 | 0.058 |
|  |  | *P-* | ***0.0445*** | *0.4944* | *0.8883* | ***0.0183*** | *0.1071* | ***0.0422*** | *0.3996* |  |  | *P* | *0.339* | *0.677* | *0.333* | *0.795* |
|  | r=10^-8^ | Exp | 3.98 | 5.54 | 0.09 | 0.93 | 2.80 | 0.99 | 0.87 |  | R=10^-8^ | Exp | 0.092 | 0.110 | 0.108 | 0.059 |
|  |  | *P-* | *0.0967* | *0.5008* | *0.8769* | ***0.0209*** | *0.0581* | ***0.0339*** | *0.3938* |  |  | *P* | *0.343* | *0.685* | *0.321* | *0.810* |
|  | r=10^-7^ | Exp | 5.91 | 5.34 | 0.08 | 0.88 | 2.58 | 0.95 | 0.83 |  | R=10^-7^ | Exp | 0.087 | 0.104 | 0.104 | 0.056 |
|  |  | *P* | *0.7147* | *0.4376* | *0.8581* | ***0.0357*** | *0.0312* | ***0.0244*** | *0.4361* |  |  | *P* | *0.274* | *0.626* | *0.278* | *0.769* |
|  |  |  |  |  |  |  |  |  |  |  |  |  |  |  |  |  |
| ***Europeans*** | |  |  |  |  | ***mean*** |  |  |  |  |  |  |  |  |  |  |
|  |  | **Obs** | **6.10** | **6.70** | **0.10** | **0.10** | **0.25** | **-0.07** | **-0.24** |  |  |  |  |  |  |  |
|  | r=0 | Exp | 6.36 | 7.23 | 0.11 | -0.04 | -0.11 | -0.03 | 0.04 |  |  |  |  |  |  |  |
|  |  | *P* | *0.3499* | *0.3271* | *0.4843* | *0.7454* | *0.7432* | *0.4221* | *0.0964* |  |  |  |  |  |  |  |
|  | r=10^-8^ | Exp | 6.59 | 7.24 | 0.11 | -0.04 | -0.30 | -0.05 | 0.03 |  |  |  |  |  |  |  |
|  |  | *P* | *0.2513* | *0.3236* | *0.4878* | *0.7537* | *0.8487* | *0.4486* | *0.0999* |  |  |  |  |  |  |  |
|  | r=10^-7^ | Exp | 8.07 | 7.20 | 0.11 | -0.02 | -1.36 | -0.03 | 0.01 |  |  |  |  |  |  |  |
|  |  | *P* | ***0.0221*** | *0.3270* | *0.4806* | *0.7277* | *0.9997* | *0.4149* | *0.1098* |  |  |  |  |  |  |  |
|  |  |  |  |  |  | ***SE*** |  |  |  |  |  |  |  |  |  |  |
|  |  | **Obs** | **1.59** | **2.52** | **0.07** | **1.14** | **2.40** | **1.26** | **0.83** |  |  |  |  |  |  |  |
|  | r=0 | Exp | 3.02 | 4.91 | 0.09 | 0.94 | 2.45 | 1.01 | 0.86 |  |  |  |  |  |  |  |
|  |  | *P* | ***0.0018*** | ***0.0061*** | *0.1809* | *0.1032* | *0.4819* | *0.0784* | *0.5023* |  |  |  |  |  |  |  |
|  | r=10^-8^ | Exp | 3.24 | 4.93 | 0.09 | 0.93 | 2.33 | 1.01 | 0.86 |  |  |  |  |  |  |  |
|  |  | *P* | ***0.0002*** | ***0.0041*** | *0.1724* | *0.0936* | *0.4022* | *0.0750* | *0.5047* |  |  |  |  |  |  |  |
|  | r=10^-7^ | Exp | 4.60 | 4.68 | 0.08 | 0.87 | 2.15 | 0.97 | 0.83 |  |  |  |  |  |  |  |
|  |  | *P* | ***0.0000*** | ***0.0072*** | *0.2257* | *0.0284* | *0.2718* | *0.0441* | *0.5437* |  |  |  |  |  |  |  |
|  |  |  |  |  |  |  |  |  |  |  |  |  |  |  |  |  |
| ***East-Asians*** | |  |  |  |  | ***mean*** |  |  |  |  |  |  |  |  |  |  |
|  |  | **Obs** | **5.30** | **5.75** | **0.09** | **0.10** | **0.24** | **0.06** | **-0.66** |  |  |  |  |  |  |  |
|  | r=0 | Exp | 6.38 | 7.25 | 0.11 | -0.04 | -0.12 | -0.03 | 0.04 |  |  |  |  |  |  |  |
|  |  | *P* | *0.0513* | *0.0817* | *0.2151* | *0.7490* | *0.7484* | *0.3504* | ***0.0025*** |  |  |  |  |  |  |  |
|  | r=10^-8^ | Exp | 6.58 | 7.23 | 0.11 | -0.04 | -0.30 | -0.03 | 0.03 |  |  |  |  |  |  |  |
|  |  | *P* | ***0.0288*** | *0.0814* | *0.2184* | *0.7527* | *0.8544* | *0.3573* | ***0.0030*** |  |  |  |  |  |  |  |
|  | r=10^-7^ | Exp | 8.01 | 7.16 | 0.11 | -0.02 | -1.33 | -0.01 | 0.01 |  |  |  |  |  |  |  |
|  |  | *P* | ***0.0016*** | *0.0872* | *0.2021* | *0.7263* | *0.9997* | *0.3737* | ***0.0013*** |  |  |  |  |  |  |  |
|  |  |  |  |  |  | ***SE*** |  |  |  |  |  |  |  |  |  |  |
|  |  | **Obs** | **1.59** | **2.61** | **0.06** | **1.08** | **2.47** | **1.25** | **1.19** |  |  |  |  |  |  |  |
|  | r=0 | Exp | 3.03 | 4.93 | 0.09 | 0.94 | 2.43 | 1.00 | 0.86 |  |  |  |  |  |  |  |
|  |  | *P* | ***0.0014*** | ***0.0071*** | *0.1213* | *0.1751* | *0.4237* | *0.0814* | *0.8601* |  |  |  |  |  |  |  |
|  | r=10^-8^ | Exp | 3.22 | 4.91 | 0.09 | 0.93 | 2.29 | 1.00 | 0.86 |  |  |  |  |  |  |  |
|  |  | *P* | ***0.0010*** | ***0.0074*** | *0.1171* | *0.1528* | *0.3193* | *0.0722* | *0.8660* |  |  |  |  |  |  |  |
|  | r=10^-7^ | Exp | 4.61 | 4.73 | 0.08 | 0.87 | 2.12 | 0.97 | 0.82 |  |  |  |  |  |  |  |
|  |  | *P* | ***0.0000*** | ***0.0100*** | *0.1458* | *0.0629* | *0.2113* | *0.0443* | *0.9166* |  |  |  |  |  |  |  |
|  |  |  |  |  |  |  |  |  |  |  |  |  |  |  |  |  |

^a^ Per generation recombination rate between adjacent base pairs

Note. Observed (Obs), mean and standard error (*SE*) of each summary statistics and indexes of neutrality tests were computed over the 20 non coding regions. The expected (Exp) values were obtained with 10^5^ simulations assuming constant (over generations) population size, which is gamma distributed (with mean *N=10,000* individuals). We simulated a finite site mutation model with a mutation rate per generation per site gamma distributed with mean ~2.5x10^-8^ and 95% confidence interval ranging from 1.47x10^-8^ to 4.03x10^-8^. We simulated regions of 1,400bp assuming (i) no recombination (r=0), (ii) a per generation recombination rate gamma distributed with a mean of ~10^-8^ (95% confidence interval ranging from 0.48x10^-8^ to 1.43x10^-8^) between adjacent base pairs and (iii) a fixed per generation recombination rate equal to 10^-7^ between adjacent base pairs.

*F*_ST_ is the *F*_ST_ computed among the sub-Saharan African, the European and the East-Asian samples; *A-E F*_ST_ is the *F*_ST_ computed between the sub-Saharan African and the European samples; *A-A F*_ST_ is the *F*_ST_ computed between the sub-Saharan African and the East-Asian samples, and *E-A F*_ST_ is the *F*_ST_ computed between the European and the East-Asian samples.
