# Supplementary material for: Formulating a Historical and Demographic Model of Recent Human Evolution Based on Resequencing Data from Noncoding Regions
Source: PLoS One. 2010 Apr 22;5(4):e10284. doi: 10.1371/journal.pone.0010284 (PMC2858654; doi:10.1371/journal.pone.0010284)
Supplement: Table S3 — Matrix of pairwise FST computed between ethnic groups. (0.04 MB DOC) [file pone.0010284.s008.doc]

**Table S3. Matrix of pairwise *F*_ST_ computed between ethnic groups**

|  | sub-Saharan Africans | | | | | Europeans | | East-Asians | |
| --- | --- | --- | --- | --- | --- | --- | --- | --- | --- |
|  | Yoruba | Ngumba | Akele | Chagga | Mozambicans | Chuvash | Danes | Han Chinese | Japanese |
| Yoruba | - | NS | NS | NS | NS | *** | *** | *** | *** |
| Ngumba | 0.00 | - | NS | NS | NS | *** | *** | *** | *** |
| Akele | 0.00 | 0.00 | - | NS | NS | *** | *** | *** | *** |
| Chagga | 0.00 | -0.01 | -0.01 | - | NS | *** | *** | *** | *** |
| Mozambicans | 0.00 | 0.01 | 0.00 | 0.00 | - | *** | *** | *** | *** |
| Chuvash | 0.12 | 0.16 | 0.14 | 0.14 | 0.12 | - | * | *** | *** |
| Danes | 0.11 | 0.15 | 0.12 | 0.13 | 0.11 | 0.01 | - | *** | *** |
| Han-Chinese | 0.11 | 0.15 | 0.14 | 0.15 | 0.13 | 0.05 | 0.06 | - | *** |
| Japanese | 0.15 | 0.18 | 0.18 | 0.18 | 0.15 | 0.04 | 0.07 | 0.03 | - |

^*^*P*<0.05, ** *P*<0.01 and *** *P*<0.001. “NS” stands for non significant.

Note. We computed pairwise *F*_ST_ between 31 Yoruba from Nigeria, 16 Ngumba from Cameroon, 16 Akele from Gabon, 32 Chagga from Tanzania, 23 Mozambicans, 23 Danes from Denmark, 24 Chuvash from Russia, 24 Han Chinese from China and 24 Japanese from Japan. For each of the 20 autosomal DNA regions, we computed pairwise *F*_ST_ using the haplotypes reconstructed using the PHASEv2 algorithm. The average pairwise *F*_ST_ is the average computed over the 20 autosomal DNA regions. In order to measure the level of genetic differentiation between populations, we evaluated the significance of each average pairwise *F*_ST_ (difference from 0) by simulating 9 samples, randomly drawn from the same population with constant effective size of 10,000 individuals (effective size was drawn from the gamma distribution described in the material and methods section). In this simulation scheme, the expected values of *F*_ST_ over 10,000 simulations is ~0.
