# Supplementary material for: Formulating a Historical and Demographic Model of Recent Human Evolution Based on Resequencing Data from Noncoding Regions
Source: PLoS One. 2010 Apr 22;5(4):e10284. doi: 10.1371/journal.pone.0010284 (PMC2858654; doi:10.1371/journal.pone.0010284)
Supplement: Table S4 — Description of the prior distributions of historical and demographic parameters simulated. (0.14 MB DOC) [file pone.0010284.s009.doc]

**Table S4. Description of the prior distributions of historical and demographic parameters simulated**

|  |  |  | **Parameter** | | | **Shape** |
| --- | --- | --- | --- | --- | --- | --- |
|  | **Parameters** |  | **mean** | **min** | **max** | **Prior** |
| **Common parameters for all models** | |  |  |  |  |  |
|  | **DNA features** |  |  |  |  |  |
|  | Mutation rate | ***μ*** | 2.5x10^-8^ | 1.3x10^-8^ | 5.05x10^-8^ | ~G |
|  | Recombination rate | ***ρ*** | 10^-8^ | 0.1x10^-8^ | 1.5x10^-8^ | ~G |
|  | **Demographic parameters** |  |  |  |  |  |
|  | Ancestral African effective population size | ***N’*** | 10000 | 500 | 40000 | ~G |
|  | Time (years) of erectus exodus from Africa | ***T_E_*** | 1.88x10^6^ | 1.25x10^6^ | 2.5x10^6^ | ~U |
|  | Time of European-Asian split | ***T_E-EA_*** | 25010 | 12520 | 37500 | ~U |
|  | Modern humans migration rate between continents | ***m*** | 2x10^-4^ | 10^-6^ | 4x10^-3^ | ND |
|  |  |  |  |  |  |  |
| **Models tested in the best fit approach (Figure 3)** | |  |  |  |  |  |
| **Common parameters to Figures 3A-C** | |  |  |  |  |  |
|  | African effective population size | ***N_A_*** | 10000 | 500 | 40000 | ~U |
|  | European effective population size | ***N_E_*** | 10000 | 500 | 40000 | ~U |
|  | East-Asian effective population size | ***N_EA_*** | 10000 | 500 | 40000 | ~U |
| **Variable parameters to Figure 3A** | |  |  |  |  |  |
|  | Replacement rate ***δ*** | | | | |  |
|  | ***δ=0*** |  | 0 | 0 | 0 | fixed |
|  | ***δ≤0.01*** |  | 0.005 | 0 | 0.01 | ~U |
|  | ***δ≤0.1*** |  | 0.05 | 0 | 0.1 | ~U |
|  | ***δ≤0.5*** |  | 0.25 | 0 | 0.5 | ~U |
|  | ***δ≥0.5*** |  | 0.75 | 0.5 | 1 | ~U |
|  | ***δ≥0.9*** |  | 0.95 | 0.9 | 1 | ~U |
|  | ***δ≥0.99*** |  | 0.995 | 0.99 | 1 | ~U |
|  | ***δ=1*** |  | 1 | 1 | 1 | fixed |
|  | Ancestral migration rate ***m_0_*** | | | | |  |
|  | ***m_0_~0*** |  | 1.75x10^-10^ | 10^-11^ | 4x10^-9^ | ~U |
|  | ***m_0_=m*** |  | 2x10^-4^ | 10^-6^ | 4x10^-3^ | ~U |
|  | ***m_0_>m*** |  | 4x10^-4^ | 2x10^-6^ | 8x10^-3^ | ~U |
|  |  |  |  |  |  |  |
| **Common parameters to Figures 3B,C** | |  |  |  |  |  |
|  | Onset of Neolithic expansion in Europe | ***t_E_*** | 8750 | 5000 | 12500 | ~U |
|  | Rate of Neolithic expansion in Europe | ***α_E_*** | 0.00255 | 0.0001 | 0.005 | ~U |
|  | Onset of Neolithic expansion in east-Asia | ***t_EA_*** | 8750 | 5000 | 12500 | ~U |
|  | Rate of Neolithic expansion in Asia | ***α_EA_*** | 0.00255 | 0.0001 | 0.005 | ~U |
| **Variable parameters to Figure 3B** | |  |  |  |  |  |
|  | Onset of African expansion, ***t_A_*** | | | | |  |
|  | ***0≤t_A_≤25K*** |  | 12,500 | 0 | 25,000 | ~U |
|  | ***25K≤t_A_≤50K*** |  | 27,500 | 25,000 | 50,000 | ~U |
|  | ***50K≤t_A_≤75K*** |  | 62,500 | 50,000 | 75,000 | ~U |
|  | Rate of African expansion ***α_A_*** | | | | |  |
|  | ***α_A_=0*** |  | 0 | 0 | 0 | fixed |
|  | ***0≤α_A_≤0.005*** |  | 0.0025 | 0 | 0.005 | ~U |
|  | ***0.005≤α_A_≤0.01*** |  | 0.0075 | 0.005 | 0.01 | ~U |
|  | ***0.01≤α_A_≤0.015*** |  | 0.0125 | 0.01 | 0.015 | ~U |
|  | ***0.015≤α_A_≤0.02*** |  | 0.0175 | 0.015 | 0.02 | ~U |
| **Variable parameters to Figure 3C** | |  |  |  |  |  |
|  | Intensity of out-of-Africa bottleneck ***β_OoA_*** | | | | |  |
|  | ***β_OoA_=1*** |  | 1 | 1 | 1 | fixed |
|  | ***1≤β_OoA_≤2*** |  | 1.5 | 1 | 2 | ~U |
|  | ***2≤β_OoA_≤20*** |  | 11 | 2 | 20 | ~U |
|  | ***20≤β_OoA_≤40*** |  | 30 | 20 | 40 | ~U |
|  | ***40≤β_OoA_≤60*** |  | 50 | 40 | 60 | ~U |
|  |  |  |  |  |  |  |

Note. ~U and ~G denote Uniformly and Gamma distributed shapes, ND (for not drawn) indicates composite parameters which result from the combination of other parameters, e.g. the Sub-Saharan population size results from the combination of *N’*, *t _A_* and *α_A_*. Times ***T*** and onsets ***t*** are expressed in number of years (generation times of 25 years). Prior distributions of the onset and the rate of African expansion, ***t_A_*** and ***α_A_***, were set to prior uniform distributions (unrealistic outcomes of sub-Saharan African populations, i.e. larger than 1 billion of individuals, were eliminated). The rates of expansion, ***α_A_***, ***α_E_*** and ***α_EA_***, are the per generation increase of population sizes expressed in percent of individuals (i.e. ***α_A_***=0.01 means the population exponentially increased by 1% of the individuals per year). The effective population sizes, ***N_A_***, ***N_E_*** and ***N_EA_***, are given in numbers of individuals. The intensity of the out-of-Africa bottleneck ***β_OoA_*** is the ratio between population sizes before and after the out-of-Africa exodus. The ancestral and modern migration rates, ***m_0_***, ***m***, are the proportion of migrants before and after the Out-of-Africa exodus. The replacement rate, ***δ***, gives the proportion of current gene lineages brought by modern humans during the out-of-Africa exodus. The mutation rate, ***μ***, is expressed in per generation per site; and ^j^ the recombination rate, ***ρ***, is expressed in per generation per pair of adjacent bases.
