# Supplementary material for: Formulating a Historical and Demographic Model of Recent Human Evolution Based on Resequencing Data from Noncoding Regions
Source: PLoS One. 2010 Apr 22;5(4):e10284. doi: 10.1371/journal.pone.0010284 (PMC2858654; doi:10.1371/journal.pone.0010284)
Supplement: Table S5 — Testing the accuracy of ABC estimations using different sets of summary statistics. (0.06 MB DOC) [file pone.0010284.s010.doc]

**Table S5. Testing the accuracy of ABC estimations using different sets of summary statistics**

|  | All summary statistics  (set 1) | | | | Removing π  (set 1 removing π) | | | | Correlated summary statistics  (set 2) | | | |
| --- | --- | --- | --- | --- | --- | --- | --- | --- | --- | --- | --- | --- |
|  | *B* | *SE* | *RMSE* | *CI_hits_* | *B* | *SE* | *RMSE* | *CI_hits_* | *B* | *SE* | *RMSE* | *CI_hits_* |
| ***t_A_*** | -0.15^b^ | 0.87 | 0.88 | 0.95 | **0.03^c^** | **0.42** | **0.42** | **0.96** | 0.19^c^ | 0.64 | 0.67 | 0.98 |
| ***α_A_*** | 0.25^c^ | 0.58 | 0.63 | 0.98 | **0.34^c^** | **0.49** | **0.59** | **0.96** | -0.11^b^ | 0.68 | 0.69 | 0.93 |
| ***N’*** | -0.01^c^ | 0.39 | 0.39 | 0.95 | **-0.04^b^** | **0.31** | **0.31** | **0.96** | -0.02^c^ | 0.39 | 0.39 | 0.95 |
| ***N_A_*** | **-2.8^b^** | **7.7** | **8.2** | **0.98** | -51.72^a^ | 126.23 | 136.41 | 0.99 | -6.12^b^ | 91.78 | 91.98 | 0.98 |
| ***T_OoA_*** | -0.03^a^ | 0.17 | 0.17 | 0.97 | -0.02^a^ | 0.17 | 0.17 | 0.99 | **-0.01^a^** | **0.17** | **0.17** | **0.98** |
| ***N_OoA_*** | 0.03^c^ | 0.28 | 0.28 | 0.98 | **0.00^b^** | **0.20** | **0.20** | **0.98** | 0.02^c^ | 0.30 | 0.30 | 0.97 |
| ***β_OoA_*** | -0.17^a^ | 0.52 | 0.55 | 0.97 | **-0.14^b^** | **0.40** | **0.42** | **0.97** | -0.21^c^ | 0.66 | 0.69 | 0.96 |
| ***N_E_*** | -0.11^b^ | 0.48 | 0.49 | 0.97 | **-0.06^c^** | **0.38** | **0.39** | **0.98** | -0.01^c^ | 0.51 | 0.51 | 0.98 |
| ***N_EA_*** | **-0.05^c^** | **0.56** | **0.57** | **0.96** | -0.05^c^ | 0.59 | 0.60 | 0.94 | -0.12^c^ | 1.06 | 1.06 | 0.92 |
| ***m*** | **-0.05^c^** | **0.31** | **0.32** | **0.97** | -0.16^c^ | 0.41 | 0.44 | 0.97 | -0.09^c^ | 0.75 | 0.76 | 0.96 |
| ***δ*** | -3x10^-6a^ | 0.03 | 0.03 | 0.90 | **-1x10^-5a^** | **0.027** | **0.027** | **0.98** | 2x10^-5a^ | 0.03 | 0.03 | 0.94 |
| ***T_E-EA_*** | -0.12^a^ | 0.36 | 0.37 | 0.95 | **0.00^c^** | **0.24** | **0.24** | **0.97** | -0.15^a^ | 0.37 | 038 | 0.95 |

^a^ mean, ^b^ median and ^c^ mode of posterior distribution

Note. The list of statistics corresponding to set 1 and set 2 can be found in supplementary Table S10. The accuracy criteria were computed by simulating 100 datasets under the best fitted model (Table 2, Figure 4A). *B* is the relative bias, *SE* is the relative standard error and *RMSE* is the relative root of mean square error. These criteria were standardized by the known parameter values. The confidence interval hits, *CI_hits_*, is the percentage of the known values falling within range of the 95% confidence interval. Our rationale for the choice of summary statistics was to first use all summary statistics, namely set 1 (sets 1 are identical between the different parameters) and we noted that removing the summary statistic π improved the results for most of the estimated parameters (set 1 removing π). We also used the summary statistics presenting the highest Pearson correlation coefficient with the parameter of interest, namely set 2 (the chosen sets of summary statistics are different among the different parameters). For each punctual estimate (mean, median and mode) and set of summary statistics, we used the simulation-based tests (see Material and Methods) in order to compute the accuracy criteria (*B*, *SE*, *RMSE* and *CI_hits_*) and to determine the most relevant set of statistics to use, namely the one minimizing the *RMSE*. By using the simulation-based tests of accuracy, we have shown there is no punctual estimate (mean, mode or median) neither set of summary statistics (sets 1 with or without π, or set 2) systematically better than others for all parameters. Therefore, we have chosen for each parameter the combinations of punctual estimates and set of summary statistics that minimized the *RMSE* (in bold), to build Table 3.
