# Supplementary material for: Formulating a Historical and Demographic Model of Recent Human Evolution Based on Resequencing Data from Noncoding Regions
Source: PLoS One. 2010 Apr 22;5(4):e10284. doi: 10.1371/journal.pone.0010284 (PMC2858654; doi:10.1371/journal.pone.0010284)
Supplement: Table S6 — ABC estimations of parameters using different sets of summary statistics. (0.05 MB DOC) [file pone.0010284.s011.doc]

**Table S6.** ABC estimations of parameters using different sets of summary statistics

|  | All summary statistics  (set 1) | | Removing π  (set 1 removing π) | | Correlated summary statistics  (set 2) | |
| --- | --- | --- | --- | --- | --- | --- |
|  | *estimate* | *95% CI* | *estimate* | *95% CI* | *estimate* | *95% CI* |
| ***t_A_*** | 37500 | 22500 - 55000 | **27500** | **20000 - 40000** | 35000 | 25000 - 50000 |
| ***α_A_*** | 0.0031 | 0 - 0.0147 | **0.007** | **0.002 - 0.016** | 0.0053 | 0.0011 - 0.0157 |
| ***N’*** | 10400 | 5900 - 19000 | **13800** | **9000 - 19800** | 10500 | 5700 - 18900 |
| ***N_A_*** | **2.35 10^7^** | **0.6x10^6^ - 1.9x10^9^** | 0.9x10^8^ | 1.75x10^7^ – 3.8x10^8^ | 0.5 10^8^ | 1.65x10^7^ - 4.15x10^8^ |
| ***T_OoA_*** | 70000 | 50000 - 92500 | 80000 | 60000 - 87500 | **60000** | **47500 - 85000** |
| ***N_OoA_*** | 3100 | 2300 - 5000 | **2800** | **2100 - 3800** | 2900 | 2100 - 4700 |
| ***β_OoA_*** | 3.6 | 1.0 - 8.9 | **5.1** | **2.6 - 8.8** | 4.1 | 1.9 - 7.2 |
| ***N_E_*** | 32800 | 20050 - 56450 | **31200** | **19600 - 52150** | 20100 | 11050 - 42000 |
| ***N_EA_*** | **14550** | **7150 - 37950** | 14450 | 6450 - 34500 | 12600 | 4250 - 39350 |
| ***m*** | **1.3 10^-5^** | **3.5x10^-6^ - 2.6x10^-5^** | 1.1x10^-5^ | 0 - 4.1x10^-5^ | 1.3x10^-5^ | 3x10^-7^ - 1.2x10^-5^ |
| ***δ*** | 0.9945 | 0.9900 - 0.9992 | **0.9949** | **0.9900 - 0.9997** | 0.9948 | 0.9901 - 0.9996 |
| ***T_E-EA_*** | 30000 | 15000 - 40000 | **22500** | **17500 - 35000** | 23000 | 15000 - 35500 |

Note. The list of statistics included in set 1 and set 2 can be found in Table S10. Times are given in years (assuming a generation time of 25 years). The estimates that proved to be the most accurate (minimizing the *RMSE*) are given in bold. These estimates are those considered in the main body of the manuscript (Table 3).
