# Supplementary material for: Formulating a Historical and Demographic Model of Recent Human Evolution Based on Resequencing Data from Noncoding Regions
Source: PLoS One. 2010 Apr 22;5(4):e10284. doi: 10.1371/journal.pone.0010284 (PMC2858654; doi:10.1371/journal.pone.0010284)
Supplement: Table S7 — Testing the influence of prior distributions on parameter estimations. (0.04 MB DOC) [file pone.0010284.s012.doc]

**Table S7.** Testing the influence of prior distributions on parameter estimations.

|  | *New prior* | | | *Estimations* | |
| --- | --- | --- | --- | --- | --- |
|  | *min* | *max* | *shape* | *estimate* | *95% CI* |
| Ancestral African effective population size ^a^ | 700 | 40000 | ~U | 15300 | 8100 - 23800 |
| Onset of African expansion ^b^ | 5000 | 75000 | ~U | 31000 | 22000 - 76000 |
| Rate of African expansion ^c^ | 0 | 0.02 | ~U | 0.0087 | 0.0005 - 0.0164 |
| Time of out-of-Africa exodus of modern humans ^d^ | 25000 | 137500 | ~U | 61000 | 35000 - 102500 |
| Replacement rate ^e^ | 0.75 | 1 | ~U | 0.93 | 0.85 - 0.99 |
| Replacement rate ^e^ | 0.9 | 1 | ~U | 0.95 | 0.9 - 0.99 |
| Migration rate ^f^ | 0 | 5x10^-3^ | ND ^f^ | 1x10^-5^ | 2x10^-6^- 2.2 x10^-5^ |

^a^ We re-sampled the 10^6^ simulations of the best fitted RAOEB model in order to obtain a flat prior distribution. Altering the shape of the priors did not alter the point estimates presented in Table 3.

^b,c^ We performed 10^5^ additional simulations of the best fitted RAOEB model by extending the priors for these parameters. We also modified the shape of the priors in order to obtain a flat prior distribution. Indeed the previous excess of low onset and rates of expansion was primarily due to the elimination of unrealistic large African effective sizes. Extending the priors and altering their shape did not alter the point estimates presented in Table 3.

^d^ We performed 10^5^ additional simulations of the best fitted RAOEB model by extending the priors for the time of out-of-Africa exodus of modern humans. Extending the prior distribution did not alter the point estimates presented in Table 3.

^e^ We performed 10^5^ additional simulations of the best fitted RAOEB model by extending the prior for the replacement rate parameter. Extending the prior altered the point estimates given in Table 3.

^f^ We performed 10^5^ additional simulations of the best fitted RAOEB model by extending the prior for the modern human migration rate between continents. Extending the prior did not alter the point estimates given in Table 3. “ND” stands for not drawn (see the legend of Table 2).

Note. We used the same estimators as those shown in Table 3. As expected, the confidence interval increased when we used a lower number of simulations and extended the priors.
