# Supplementary material for: Formulating a Historical and Demographic Model of Recent Human Evolution Based on Resequencing Data from Noncoding Regions
Source: PLoS One. 2010 Apr 22;5(4):e10284. doi: 10.1371/journal.pone.0010284 (PMC2858654; doi:10.1371/journal.pone.0010284)
Supplement: Table S8 — Testing the influence of prior distributions for some parameters on the estimation of other parameters. (0.05 MB DOC) [file pone.0010284.s013.doc]

**Table S8.** Testing the influence of prior distributions for some parameters on the estimation of other parameters

|  | Removing π  (set 1 removing π) | |
| --- | --- | --- |
|  |  | |
|  | Flat prior for ***N’*** | |
|  | *Estimate* | *95% CI* |
| ***t_A_*** | 27500 | 17500 - 42500 |
| ***α_A_*** | 0.008 | 0.000 - 0.017 |
| ***N’*** | **15300** | 8100 - 23800 |
| ***2N_A_*** | 2.0x10^8^ | 0.55x10^6^ - 1.7x10^9^ |
| ***T_OoA_*** | 71000 | 49900 - 96400 |
| ***N_OoA_*** | 2900 | 2100 - 3900 |
| ***β_OoA_*** | 6.2 | 2.0 - 11.7 |
| ***N_E_*** | 31100 | 18100 - 51100 |
| ***N_EA_*** | 15550 | 5650 - 33750 |
| ***m*** | 1.07x10^-5^ | 3.97x10^-6^ - 2.56x10^-5^ |
| ***δ*** | 0.995 | 0.99 - 1 |
| ***T_E-EA_*** | 23600 | 15700 - 33500 |
|  |  |  |
|  | Extended prior for *δ* | |
|  | *Estimate* | *95% CI* |
| ***T_OoA_*** | 72300 | 52400 - 93200 |
| ***N_OoA_*** | 2800 | 2100 - 3800 |
| ***β_OoA_*** | 6.8 | 2.2 - 14.5 |
| ***N_E_*** | 24700 | 14100 - 39500 |
| ***N_EA_*** | 16700 | 7050 - 33900 |
| ***m*** | 1.64x10^-5^ | 7.63x10^-6^ - 3.65x10^-5^ |
| ***δ*** | **0.95** | **0.91 - 0.99** |
| ***T_E-EA_*** | 19771 | 10733 - 30165 |

Note. The estimations (using set 1 of summary statistics removing π, and the mean of posterior distribution) were performed using a flat prior for the ancestral African population size (N’). Changing the shape of prior distribution for this central parameter did not alter the estimation of the other parameters, with the exception of the time of Out of Africa exodus which nevertheless kept values in the range of those generally provided in the literature (~71,000 [49,000 to 96,000] years ago). These estimations were also performed using an extended and flat prior for *δ* (replacement rate). In this case, accounting for a larger uncertainty for this parameter did not alter the estimation of the other parameters, with the exception of the time of Out of Africa exodus ,which nevertheless kept values in the range of those generally provided in the literature (~72,000 [52,000 to 93,000] years ago).
