# Supplementary material for: Formulating a Historical and Demographic Model of Recent Human Evolution Based on Resequencing Data from Noncoding Regions
Source: PLoS One. 2010 Apr 22;5(4):e10284. doi: 10.1371/journal.pone.0010284 (PMC2858654; doi:10.1371/journal.pone.0010284)
Supplement: Table S9 — Testing the influence of the models of human dispersals out of Africa used, on parameter estimations. (0.04 MB DOC) [file pone.0010284.s014.doc]

**Table S9.** Testing the influence of the models of human dispersals out of Africa used, on parameter estimations.

|  | Estimation^a^ | |
| --- | --- | --- |
|  | *Estimate* | *95% CI* ^b^ |
| ***t_A_*** | 30000 | 20000 - 45000 |
| ***α_A_*** | 0.0056 | 0 - 0.0163 |
| ***N’*** | 12200 | 7400 - 19100 |
| ***N_A_*** | 9.2x10^7^ | 2.7x10^5^ - 7.2x10^8^ |
| ***T_OoA_***^c^ | 67500 | 50000 - 90000 |
| ***N_OoA_*** | 2850 | 2000 - 4050 |
| ***β_OoA_*** | 4.2 | 2.6 - 8 |
| ***N_E_*** | 24600 | 13700 - 42000 |
| ***N_EA_*** | 13200 | 3000 - 32500 |
| ***m*** | 1.2x10^-5^ | 6x10^-6^ - 3.4x10^-5^ |
| ***δ*** | 0.9951 | 0.9902 - 0.9998 |
| ***T_E-EA_*** | 22500 | 12500 - 32500 |

^a^ For each parameter estimation, we report the values obtained using the set of summary statistics giving the best accuracy (parameters in bold in Table S5); ^b^ 95% Bayesian confidence interval; ^c^ This parameter was estimated considering simulations performed under models A and B only (Figures 4A and 4B), because models C and D (Figures 4C and 4D) consider two dispersals out of Africa occurring at different times.
