# Supplementary material for: Formulating a Historical and Demographic Model of Recent Human Evolution Based on Resequencing Data from Noncoding Regions
Source: PLoS One. 2010 Apr 22;5(4):e10284. doi: 10.1371/journal.pone.0010284 (PMC2858654; doi:10.1371/journal.pone.0010284)
Supplement: Table S10 — List of summary statistics used. (0.05 MB DOC) [file pone.0010284.s015.doc]

**Table S10. List of summary statistics used**

|  | ***Model-testing*** | | | ***ABC*** | |
| --- | --- | --- | --- | --- | --- |
| **Summary statistics** | **MRE vs RAO** | **Sub-Saharan**  **expansion** | **Non-African bottleneck** | ***SET1*** | ***SET2 (T_OoA_)*** |
|  |  |  |  |  |  |
|  |  |  |  |  |  |
| Global *F_ST_* | **+** | **+** | **+** | **+** | **+** |
| Pair wise *F_ST_* | **+** |  |  | **+** |  |
| **Merging samples** |  |  |  |  |  |
| *K* | **+** |  |  | **+** | **+** |
| *S* | **+** |  |  | **+** | **+** |
| *π%* | **+** |  |  | **+** |  |
| *D* | **+** |  |  | **+** |  |
| **In each sample** |  |  |  |  |  |
| *K* |  | **+** | **+** | **+** | **+** |
| *S* |  | **+** | **+** | **+** | **+** |
| *π%* |  | **+** | **+** | **+** |  |
| *D* |  | **+** | **+** | **+** |  |
|  |  |  |  |  |  |
